# Dynamics of career attractiveness and preferences among Swiss medical students: an observational study at the end of the master’s program

**DOI:** 10.1080/10872981.2025.2592434

**Published:** 2025-11-30

**Authors:** Stefania Di Gangi, Markus Inauen, Stefan Markun, Oliver Senn

**Affiliations:** a Institute of Primary Care, University of Zurich, University Hospital Zurich, Zurich, Switzerland

**Keywords:** Undergraduate medical education, medical specialties, student perception, career interests, career preferences

## Abstract

Medical career preferences are in focus because the future medical workforce should align with society’s needs. The study investigated how medical students’ perceptions of the attractiveness of various clinical and non-clinical career options evolved as they approached the end of medical school, and which factors might influence their career choices. This was a cross-sectional online survey of medical students who completed the master’s program in spring 2025 in different medical education tracks at different Swiss universities. The survey included both Likert-scaled and open-ended questions. Flow diagrams were used to depict changes in attractiveness throughout medical school. Network visualization mapped the connections between the most important career determinants. Regression analysis assessed the factors associated with career preferences. Among 364 medical students included, the most attractive careers at the end of medical school were the specialized disciplines of inpatient care (37%) and outpatient care (20%). These specialties were preferred due to interest in surgery or specialization. During the master’s program, attractiveness of general practice, specialized outpatient care, and specialized inpatient care increased while the attractiveness of outpatient gynecology/pediatrics and inpatient general internal medicine decreased. Career characteristics perceived to be the most important determinants of career choice were primarily performing medical activities, part-time work, and relationships with patients. The most prevalent factors favoring career decisions were experience during the elective year (91%) and clinical courses with patient contact during the studies (70%). Students who found a career more attractive during their studies were more likely to prefer that career at the end of medical school. Career preferences at the end of medical school were associated with specific factors. Among these factors, the most significant was the perceived attractiveness of the career during medical education. This emphasizes the importance of medical education in shaping students' dynamic and multifaceted career decisions.

## Introduction

Medical students' career preferences often change during medical school and change in different ways at different medical schools [1–6]. Only one in four medical students who start medical school with a career in mind may choose the same specialty by the time they graduate [7]. There is also evidence that some students enter medical school without a clear intention to become doctors [8]. Choosing a medical career is a complex personal decision influenced by many factors [8–11]. Informal and hidden curricula at medical school can also influence career choice [12,13].

Graduates' career choices are not well aligned with society's needs [14]. Therefore, monitoring medical students' career interests and understanding the factors that influence their specialty choice can help medical education institutions to counteract workforce shortages [15–17]. At the same time, action is also needed to increase the participation of medical doctors in research [18]. There is also a need to investigate medical students' interest in non-medical careers to better understand their career planning needs and the factors that influence their career choices [8].

In light of these considerations and research needs, the aim of this study was to provide further insights into medical students' career preferences and choices. The present study investigated the evolution of medical students' perceptions of the attractiveness of various clinical and non-clinical career options as they approached the conclusion of their medical education. Moreover, the investigation encompassed an analysis of the students' career preferences at the conclusion of their studies and the factors associated with these preferences. The study also examined the factors that may potentially influence the students' career choices.

## Materials and methods

### Context, design and participants

In Switzerland, undergraduate medical education, as generally defined [19], lasts six years, including both bachelor and master programs of three years each. The bachelor's program provides a solid foundation in basic medical sciences and includes introductory clinical skills training. In the master's program, students receive clinical training and complete rotations in various medical specialties.

We conducted a cross-sectional survey of medical students at the end of their master’s program in spring 2025. The students surveyed were enrolled in different Swiss universities: Zurich, St. Gallen, Lucerne, Basel, and Lugano. Previous surveys have been conducted during the bachelor's program [20,21].

Participation in the survey was voluntary. Students were invited via online chat groups, which had been established within the respective master's programs, and were reminded of the invitation via a follow-up message.

### Questionnaire

The online questionnaire was in German on the RedCap® survey platform. It was self-administered and specifically designed for the purposes of the study after a review of the literature. To ensure clarity and accuracy, the final-draft was pilot-tested with ten students from the target population. The questionnaire mainly covered two topics: career attractiveness/preferences, and determinants of career choice. The first topic considered specific and aggregated medical career options like general practice, outpatient gynecology/pediatrics, other specialized outpatient disciplines, inpatient care disciplines, research and non-clinical careers. The second topic examined job characteristics (e.g., income, security, part-time work, relationships with patients) which have been identified as determinants of a medical career [20–23] and other factors (e.g., friends’/family’s opinions, clinical courses, mentoring/advice from doctors, COVID−19 pandemic) that might positively or negatively influence a career choice [3,12,13,24,25]. The survey included a mix of question formats, such as multiple choice, 5-point Likert scale items, dropdown lists, and open-ended questions. A copy of the translated questionnaire in English was reported as supplementary information (Supplementary Material 1).

### Study outcomes

Study outcomes were expressed as percentages of students who:

1) found a proposed career option attractive at the end of medical school;
2) found a proposed career as the most attractive (career preference) or the least attractive at the end of medical school;
3) increased, decreased or did not change their attractiveness to a career during medical education;
4) rated the proposed job characteristics as important and selected the three most important ones;
5) reported being influenced by the different factors (e.g., society, family) when choosing their career.

Additionally, we gained insight into the reasons for rating a career as the most or least attractive. We also identified factors associated with career preferences.

### Data analysis

Student characteristics and measurements were described as numbers and percentages, n(%), for categorical or binary variables and as mean (standard deviation (SD)) or median [interquartile range (IQR)], as appropriate, for continuous variables. Available case analysis was performed and the number of non-missing responses was reported for each question.

The results obtained from responses using a five-point Likert scale were presented in the form of a diverging bar chart. Flow diagrams (alluvial plots) [26] were used to depict changes in the attractiveness of each career during medical school, and chi-squared tests were used to compare these changes during the bachelor’s and master’s programs. Network visualization [27] was used to represent the relationship between the most important career determinants.

Logistic regression analysis, multivariable, was carried out to identify factors associated with career preferences. The results of regression analyzes were reported as odds ratios (OR) with 95% confidence intervals (CI). A p ≤ 0.05 was used to determine statistical significance.

Qualitative analysis of the open-ended questions was performed using a framework analysis [28]. Tags, assigned by the researcher from student texts, were used as conceptual codes and visualized through tag cloud figures. This methodology was used in a previous study on medical education [29].

Statistical analysis was performed using the R software (version 4.5.0) [30].

## Results

### Participants

Of the 649 students who completed the surveyed master’s programs in 2025, 383 responded to the survey. Nineteen students were excluded from the analysis because they did not answer any of the questions related to the study outcomes. Therefore, the number of participants was 364, for a response rate of 56%. Participants were predominantly female 226 (68%) with a mean age of 25.77 (SD = 1.96) years. Thirty students did not respond to the gender question, and three identified as diverse.

### Career attractiveness, preferences and dynamics

**Figure 1.**
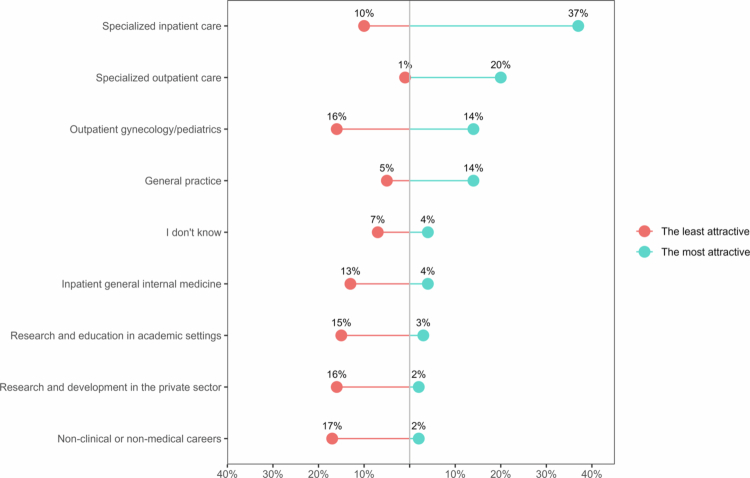
Career preferences of 364 students at the end of the master’s program in Swiss medical education. Notes: The percentages of students who selected each career as the most attractive in response to question 4 (see Supplementary Material 1), were reported on the right side of the plot. The percentages of students who selected each career as the least attractive in response to question 6, were reported on the left side of the plot.

The career options that the majority of students perceived as attractive at the end of the master's program were specialized inpatient care and outpatient care (59%); general practice followed with 51% (Supplementary Material 2; Figure S1). However, when tasked with identifying the career preference, 37% of students chose specialized inpatient care, 20% chose specialized outpatient care, and only 14% chose general practice (Figure 1). Conversely, least attractive were non-clinical or non-medical careers (17%), research in the private sector (16%), and outpatient gynecology/pediatrics (16%).

**Figure 2.**
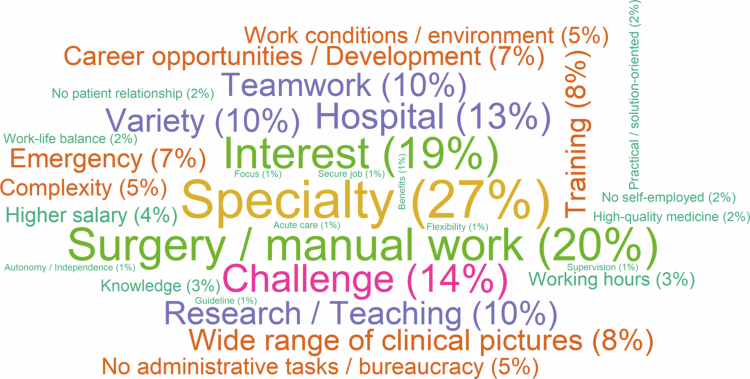
Tag cloud of reasons for preferring a career in specialized inpatient care, the most attractive career option selected by 135 students. Notes: Tags were assigned based on the responses to the questions 5 (see Supplementary Material 1) regarding the selection of specialized inpatient care in question 4. The percentage for each tag was reported in relation to the 118 students answering the question. More frequent tags appeared larger than the less frequent ones.

A career in specialized inpatient care was preferred for the specialization (27%), the surgery or manual work (20%) and the interest (19%) (Figure 2). The reasons for preferring other career options were summarized in Supplementary Material 2; Figure S2. Examples of conceptual codes and students’ quotes from open-ended questions, were reported in Supplementary Material 2; Tables S1 and S2. Research and other non-clinical or non-medical careers were considered the least attractive, mainly due to the absence of clinical/medical work, a lack of interest, and the absence of patients (Supplementary Material 2; Figure S3).

**Figure 3.**
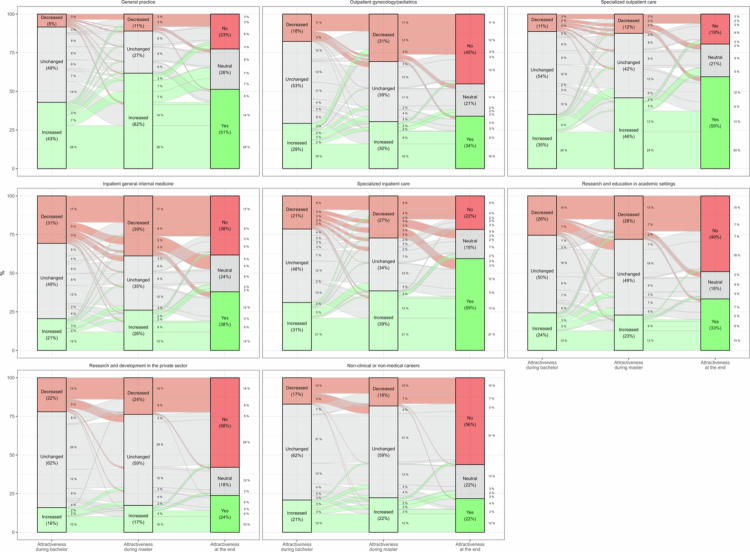
Alluvial plot of the students’ dynamics of the attractiveness of each career option during medical school. Notes: The first and second dimensions were the changes in attractiveness during the bachelor’s and master’s program, respectively (see questions 8 and 9 in Supplementary Material 1). The third dimension was the career’s definite attractiveness at the end of medical education (question 3) coded as follows: yes = very attractive/rather attractive, neutral = neutral, no = rather unattractive/very unattractive. A total of 345 students answered all three questions. Percentages of the students in each stratum and flow were reported when percentages were above 1%.

The dynamics of the attractiveness of each career option, from the bachelor’s program to the end of master’s program, were illustrated in Figure 3. A higher percentage of students increased their attractiveness to general practice during the master’s program than during the bachelor’s program (62% vs. 43%, *p* < 0.001). A similar trend was noted for specialized outpatient care (46% vs. 35%, *p* = 0.006), and specialized inpatient care (39% vs. 31%, *p* = 0.002). Conversely, a higher percentage of students decreased their attractiveness to outpatient gynecology/pediatrics during the master’s program than during the bachelor’s program (31% vs. 18%, *p* < 0.001). A similar trend was noted for inpatient general internal medicine (39% vs. 31%, *p* = 0.001). The attractiveness of other career options (research and non-clinical careers) remained unchanged. At the end of the master’s program, most students who were attracted to a specific career reported that this career became more attractive to them during both the bachelor's and master's programs. The transitions from attractiveness during the bachelor’s and master’s programs to career preference were illustrated in Supplementary Material 2; Figure S4.

**Figure 4.**
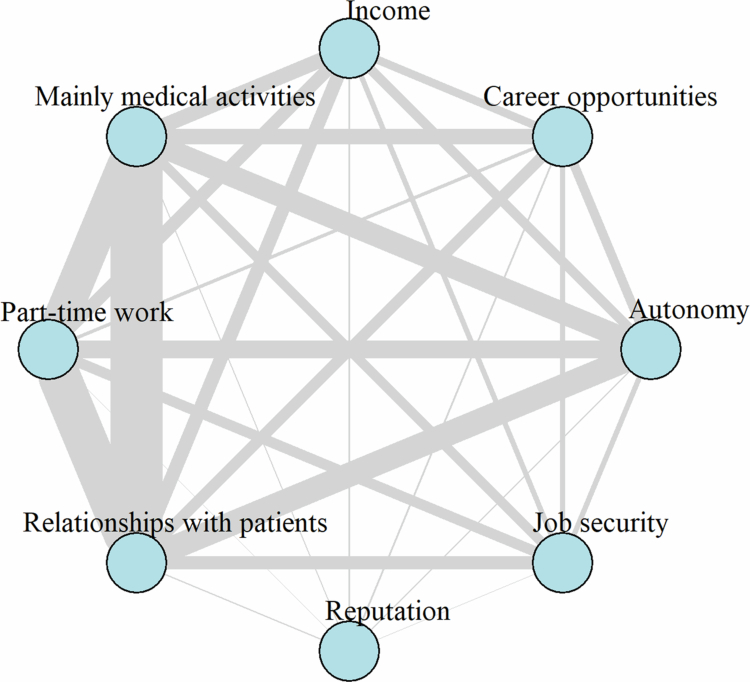
Network visualization of the interplay of the three most important career determinants at the end of the master’s program in Swiss medical education. Notes: The three most important determinants were the answers, *n* = 343, to question 11 (see Supplementary Material 1). The represented network was weighted und undirected. Thicker, denser colored edges indicated stronger relationships between determinants, depending on the number of students who chose them. ‘Primarily performing medical activities’ was abbreviated as ‘mainly medical activities’.

### Determinants of career choice

The career determinants that the largest percentage of students perceived as important were primarily performing medical activities (84%), relationships with patients (80%), and job security (80%) (Supplementary Material 2; Figure S5). When representing the three most important career determinants (Figure 4), the strongest relationship between primarily performing medical activities, relationships with patients, and part-time work emerged. In fact, these three elements were more commonly selected together (by 21% of all students).

**Figure 5.**
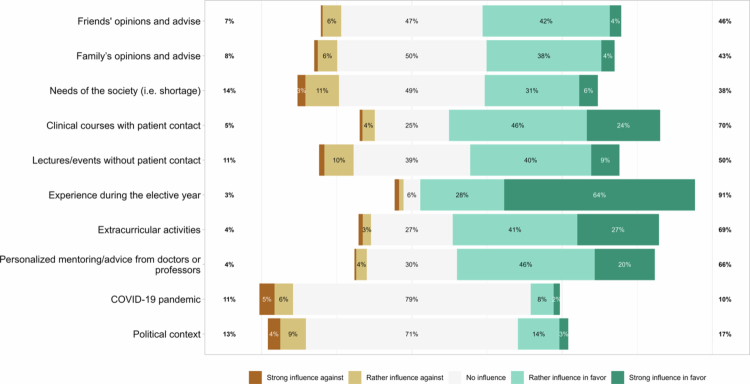
Factors favoring or hindering a career choice at the end of the master’s program in Swiss medical education: overall results. Notes: The results are relative to the responses, *n* = 344, to question 12 (see Supplementary Material 1). The right side showed the percentages of positive responses (rather influence in favor/strong influence in favor). In the middle were the percentages of neutral responses (no influence), and on the left were the percentages of negative responses (strong influence against/rather influence against).

The most prevalent factors favoring a career choice were experience during the elective year (91%), clinical courses with patient contact during the studies (70%), and extracurricular activities (69%) (Figure 5). The most common factors hindering a career choice were needs of society (e.g., shortage in the specialty) (14%) and the political context (13%). In addition, the negative impact of the pandemic on the choice of inpatient general internal medicine and on an academic career appeared to be considerable (Supplementary Material 2; Figure S6).

### Factors associated with career preferences

Table 1 reported the results of the multivariable regression analysis of the factors associated with career preferences at the end of the master’s program. An increased career attractiveness during medical education was the factor with the strongest positive association with nearly all career options. Age and selecting patient relationships as one of the three most important career determinants were positively associated with a preference for general practice (OR (95%CI): 1.24 (1.06−1.46) and 3.59 (1.52−9.98), respectively), but negatively associated with a preference for research or other non-clinical or non-medical careers (0.67 (0.44−0.96) and 0.27 (0.09−0.74), respectively). Female gender was positively associated with a preference for gynecology/pediatrics, 4.37 (1.41−19.19). Working part-time, autonomy and income, as chosen among the most important career determinants, were positively associated with a preference for specialized outpatient care and negatively associated with a preference for specialized inpatient care.

**Table 1.** Multivariable logistic regression analysis of the factors associated with a career preference at the end of the master's program in Swiss medical education.

	General practice *n* = 338			Outpatient gynecology/pediatrics *n* = 338			Specialized outpatient care *n* = 338			Inpatient general internal medicine *n* = 345			Specialized inpatient care *n* = 345			Research/Non-medical careers (grouped together) *n* = 338		
Factors	Odds Ratio	Confidence Interval	p	Odds Ratio	Confidence Interval	*p*-value	Odds Ratio	Confidence Interval	*p*-value	Odds Ratio	Confidence Interval	*p*-value	Odds Ratio	Confidence Interval	*p*-value	Odds Ratio	Confidence Interval	*p*-value
Age (years)	1.24	1.06−1.46	**0.009**													0.67	0.44−0.96	**0.044**
Female gender [Yes] (reference category: No)				4.37	1.41−19.19	**0.022**	0.62	0.33−1.18	0.142									
Attractiveness increased [Yes] (reference category for each variable: No, non-increased)																		
During bachelor’s program	1.31	0.66−2.64	0.451				2.41	1.28−4.56	**0.007**	2.59	0.79−9.73	0.132	2.01	1.13−3.57	**0.017**	2.45	0.75−9.18	0.155
During master’s program	5.32	2.08−16.49	**0.001**	15.11	6.72−38.82	**<0.001**	3.07	1.60−6.08	**0.001**	13.16	2.94−93.27	**0.002**	4.24	2.46−7.37	**<0.001**	3.29	0.98−13.42	0.069
Career determinants rated among the three most important (reference category for each variable: No, not rated as the most important)																		
Patient relationships [Yes]	3.59	1.52−9.98	**0.007**	2.42	1.00−6.51	**0.061**							0.57	0.32−1.02	0.061	0.27	0.09−0.74	**0.013**
Career opportunities [Yes]	0.26	0.06−0.80	**0.037**															
Working part-time [Yes]							2.56	1.35−5.00	**0.005**				0.38	0.22−0.66	**0.001**			
Autonomy [Yes]							1.89	1.01−3.58	**0.047**				0.45	0.25−0.80	**0.008**			
Income [Yes]							4.44	2.33−8.61	**<0.001**				0.53	0.28−0.99	**0.050**			
Primarily performing medical tasks [Yes]																0.20	0.06−0.56	**0.004**

Notes: The selection of variables for multivariable models was based on a stepwise backward approach, starting from a full model including all variables (student characteristics, ratings of the most important career determinants, changes in career attractiveness during medical school) and excluding them using the Akaike information criterion (AIC).

## Discussion

The present study contributes to the existing body of knowledge in medical education by offering insights into medical students' career preferences and the determinants of their career choices. The dynamics of students' attractiveness to different medical and non-medical career options during their undergraduate medical education were depicted. The reasons for considering each career option as the most or least attractive were explored. Visual methods were employed to conceptualize the interplay between career determinants. The study assessed factors associated with career preferences and provided a description of how other factors, such as the pandemic or hidden curricula, could potentially influence career choices.

### Comparison with literature

During the master’s program, general practice, and specialized outpatient and inpatient care disciplines became more attractive. These results confirmed the trend already observed in specialized outpatient and inpatient care careers [21]. They also showed that the attractiveness of general practice increased with exposure to the profession during the master’s program, which is consistent with other studies [29,31–36]. Conversely, outpatient gynecology/pediatrics and inpatient general internal medicine became less attractive. The trend in outpatient gynecology/pediatrics differs from the one observed during the bachelor’s program [21], but it is in line with a recent study [37]. Further investigation is needed to determine whether this is a recent shift or simply confirmation that pediatrics and obstetrics-gynecology are the least stable medical specialties [4,6]. Unlike other studies, we used flow diagrams to depict the dynamics of students’ attractiveness to different medical and non-medical career options during their undergraduate medical education, resulting in novel findings. However, a deeper understanding of these dynamics would require further research, including the analysis of students’ characteristics, such as personality and innate interest in a specialty, that may have contributed to them.

Regarding career preferences, our results are consistent with other research identifying surgical disciplines as the most popular definite career choice, whether alone [24,38–43] or together with anesthesiology [44]. Although 51% of students considered general practice attractive, only 14% identified it as their preferred career. This result is consistent with previous research [25].

The attractiveness and preference for research careers were substantially lower compared with a previous study where one-third of medical students plan to pursue a career in medical research after graduation [18] but however in line with other studies [45,46]. Further research is also needed on this topic, as there is limited literature [20,21] examining research as a career option compared to other medical careers. In our study, the percentage of undecided students was low (4%), which is consistent with previous findings that the proportion of undecided students decreases as students progress through their degree courses [44,47]. While only a 2% of the students reported a preference for non-clinical or non-medical careers, 22% found these options attractive. This result needs further investigation, as it could mean that one in five students may enroll in medical school without clear intentions to join the medical workforce, consistently with a recent study [8].

The three most important career determinants were primarily performing medical activities, building relationships with patients and working part-time. These findings are consistent with those of a previous study which found that lifestyle considerations are among the top three factors influencing specialty choice [48], and that students generally desire regulated working hours and a good work-life-balance [21,24,42–44]. However, unlike other studies, we used network analysis to model the interplay of the three most important careers determinants, resulting in novel findings.

The most prevalent factors favoring a career choice were experience during the elective year, clinical courses with patient contact during the studies, and extracurricular activities. These findings are consistent with previous studies [3,23,25,36,37,44,49,50]. On the other hand, one of the most common factors hindering a career choice was the political context. In particular, it represents the most relevant factor hindering the choice of general practice, which confirms previous findings [13,21]. In addition, the negative effects of the pandemic were reported to have a greater impact on the inpatient general internal medicine and on an academic career. These results are consistent with a previous study [51].

The results of the qualitative analysis could not be directly compared with those in the literature, with the exception of a few investigations [13,22,23] that utilized qualitative methodologies to discern the rationales for either being or not being interested, or for opting or not for a career in general practice. However, the main reasons for finding specialized inpatient care the most attractive career, such as surgery or manual work, and the main reasons for finding it the least attractive, such as workload, working conditions and a preference for work-life balance, are among the factors influencing the career choice that previous research identified [24,38,39]. The gained insights into academic careers and, in particular, the students’ reported lack of preparation for research during their studies, as well as a lack of knowledge regarding research in the private sector, are also evidenced in previous research [18,52]. Our insights are also consistent with previous findings regarding the factors influencing the choice or preference for gynecology/pediatrics [37,53].

Furthermore, we assessed the factors associated with career preferences. Previous studies also identified age [54–56] and the importance of patient relationships [13,21,23,29,35,57–59] as positively associated with a career in general practice, while career development opportunities [23,48,57,59] were identified as negatively associated; female gender as positively associated with a preference for gynecology/pediatrics [21,39,43,44,59–66]; working part-time, autonomy and income as positively associated with a preference for specialized outpatient care and negatively associated with a preference for specialized inpatient care [23,24,44]. Distinct from other studies, the career preference model in our study was adjusted to account for the career's attractiveness during medical school. This factor demonstrated the strongest positive association with nearly all career options.

### Limitations

The main limitation of this study is that, in order to study students’ dynamics using anonymous data, we asked students to assess the attractiveness of each career option at different time points: during the bachelor’s program, during the master’s program and at the end of the master’s program. Therefore, our data may be subject to recall bias. Meanwhile, our survey allowed students to reflect on their entire medical education journey, offering a more insightful and comprehensive overview than assessments at individual points in time would provide.

Second, we did not perform subgroup analysis within the medical education tracks. This would be the focus of another study. Moreover, we assumed that students’ career intentions were the ones selected as ‘the most attractive’ from the list. However, these intentions may differ from the career plans immediately following graduation and may not reflect future career objectives.

Finally, we did not have information on socio-economic variables, parental occupation, academic abilities and personality traits that may influence career choices [8,35,43,48]. Therefore, our multivariate analysis may not have captured all confounding factors.

### Implications for research and practice

The main message of this study is that if a medical career is found more attractive by students during undergraduate medical education, that career is more likely to be preferred by them at the end of medical school. Moreover, experience during the elective year, clinical courses with patient contact, and extracurricular activities during the studies appear to be the most relevant factors favoring a career choice. This reinforces the role of medical education in shaping students’ career preferences and choices and suggests that, during their undergraduate studies, students should be introduced to all medical specialties and provided with early and ongoing clinical exposure to develop a realistic understanding of the various career options available to them. Though this study evidenced the impact of the exposure during the master’s program on increasing the attractiveness of a career in general practice, only approximately one in ten students reported a preference for the career, which is in accordance with previous estimates [4,13]. Therefore, addressing workforce shortages in general practice remains a major concern.

Our findings also suggest enhancing the role of research in the medical curriculum. Research careers were, in fact, perceived by students as being no different from any other non-clinical and non-medical option.

## Conclusions

This study provided valuable insights into the attractiveness to medical students of various clinical and non-clinical career options at the conclusion of their master's programs. An association was identified between career preferences and specific factors. However, the most significant factor impacting career preferences overall was the attractiveness of the career during medical education. This emphasizes the importance of medical education in shaping students' dynamic and complex career decisions.

## Supplementary Material

Supplementary Material Supplementary Material 1.

Supplementary Material Supplementary Material 2.

## Data Availability

The datasets used and/or analyzed during the current study are available from the corresponding author on reasonable request.
