# Supplementary material for: Dynamics of career attractiveness and preferences among Swiss medical students: an observational study at the end of the master’s program
Source: Med Educ Online. 2025 Nov 30;30(1):2592434. doi: 10.1080/10872981.2025.2592434 (PMC12667345; doi:10.1080/10872981.2025.2592434)
Supplement: Supplementary Material — 1. [file ZMEO_A_2592434_SM4857.docx]

**Study on educational background, career interest, professional values and future plans among medical students in Switzerland**

Thank you for completing this short questionnaire (approx. 10 minutes of your time). The study is being conducted at several universities in Switzerland. Your participation is voluntary and anonymous.

The results will help you develop your educational path. Please answer the survey to the best of your knowledge and belief. Please read the questions and answer options carefully before you answer.

Your answers will be treated confidentially and published in a summarized form (without drawing conclusions about individual participants) in a report.

**As a thank you, CHF 10 will be donated to the university union.**

Thank you for your participation!

***[Checkbox]** *I agree.*

**1) Information about the degree program**

***1. Which university or educational pathway did you originally apply to (when registering for the numerus clausus aptitude test)?**

- University of Zurich
- University of Zurich (Lucerne Track)
- University of Zurich (St. Gallen Track)
- ETH Zurich
- University of Basel
- University of Basel-USI
- University of Bern
- University of Fribourg
- Other (please specify)

***2. Which university or educational pathway were you enrolled in during your Bachelor's degree?**

- University of Zurich
- University of Zurich (Lucerne Track)
- University of Zurich (St. Gallen Track)
- ETH Zurich
- University of Basel
- University of Basel-USI
- University of Bern
- University of Fribourg
- Other (please specify)

**2) Attractiveness of career options**

***3. How attractive are the following career options as possible career goals for you at present**?

| **Occupational field** | **Very unattractive** | **Rather unattractive** | **Neutral** | **Rather attractive** | **Very attractive** |
| --- | --- | --- | --- | --- | --- |
| Primary care in a general practice | ○ | ○ | ○ | ○ | ○ |
| Primary care in a pediatrics or gynecology practice | ○ | ○ | ○ | ○ | ○ |
| Specialized medicine in a practice (e.g. cardiology, gastroenterology, rheumatology, etc.) | ○ | ○ | ○ | ○ | ○ |
| General internal medicine in a hospital (diseases of the internal organs without surgical activities) | ○ | ○ | ○ | ○ | ○ |
| Specialized medicine in a hospital (including surgical specialties) | ○ | ○ | ○ | ○ | ○ |
| Research and teaching at a university/college (academic career) | ○ | ○ | ○ | ○ | ○ |
| Research and development in the private sector | ○ | ○ | ○ | ○ | ○ |
| Other non-clinical or non-medical professions | ○ | ○ | ○ | ○ | ○ |

***4. What is currently the most attractive career goal for you?**
*(Drop-down list: Primary care in a general practice, Primary care in a pediatric or gynecology practice, Specialized medicine in a practice, General internal medicine in a hospital, Specialized medicine in a hospital, Research and teaching at a university/college, Research and development in the private sector, Other non-clinical or non-medical professions, I don't know).*

**5 (Optional). Why is this currently the most attractive career goal for you?** *(free text field)*

***6. What is currently the least attractive career goal for you?**

*(Drop-down list: Primary care in a general practice, Primary care in a pediatric or gynecology practice, Specialized medicine in a practice, General internal medicine in a hospital, Specialized medicine in a hospital, Research and teaching at a university/college, Research and development in the private sector, Other non-clinical or non-medical professions, I don't know)*

**7 (Optional). Why is this currently the least attractive career goal for you?** *(free text field)*

***8. How did your attractiveness of the following career options develop during your Bachelor's degree program (between the start of your studies and the completion of your Bachelor's degree)?**

| **Occupational field** | **decreased** | **unchanged** | **increased** |
| --- | --- | --- | --- |
| Primary care in a general practice | ○ | ○ | ○ |
| Primary care in a pediatrics or gynecology practice | ○ | ○ | ○ |
| Specialized medicine in a practice (e.g. cardiology, gastroenterology, rheumatology, etc.) | ○ | ○ | ○ |
| General internal medicine in a hospital (diseases of the internal organs without surgical activities) | ○ | ○ | ○ |
| Specialized medicine in a hospital (including surgical specialties) | ○ | ○ | ○ |
| Research and teaching at a university/college (academic career) | ○ | ○ | ○ |
| Research and development in the private sector | ○ | ○ | ○ |
| Other non-clinical or non-medical professions | ○ | ○ | ○ |

***9. How did your attractiveness of the following career options develop during your Master's degree program?**

| **Occupational field** | **decreased** | **unchanged** | **increased** |
| --- | --- | --- | --- |
| Primary care in a general practice | ○ | ○ | ○ |
| Primary care in a pediatrics or gynecology practice | ○ | ○ | ○ |
| Specialized medicine in a practice (e.g. cardiology, gastroenterology, rheumatology, etc.) | ○ | ○ | ○ |
| General internal medicine in a hospital (diseases of the internal organs without surgical activities) | ○ | ○ | ○ |
| Specialized medicine in a hospital (including surgical specialties) | ○ | ○ | ○ |
| Research and teaching at a university/college (academic career) | ○ | ○ | ○ |
| Research and development in the private sector | ○ | ○ | ○ |
| Other non-clinical or non-medical professions | ○ | ○ | ○ |

**3) Determining factors in career choice**

***10. How important are the following aspects for you when choosing a career?**

| **Aspect** | **Not at all important** | **Rather not important** | **Neutral** | **Rather important** | **Very important** |
| --- | --- | --- | --- | --- | --- |
| Financial income | ○ | ○ | ○ | ○ | ○ |
| Reputation | ○ | ○ | ○ | ○ | ○ |
| Possibility of part-time work | ○ | ○ | ○ | ○ | ○ |
| Doctor-patient relationship | ○ | ○ | ○ | ○ | ○ |
| Primarily performing medical activities | ○ | ○ | ○ | ○ | ○ |
| Career opportunities | ○ | ○ | ○ | ○ | ○ |
| Autonomy at work | ○ | ○ | ○ | ○ | ○ |
| Job security | ○ | ○ | ○ | ○ | ○ |

***11. Please select the three most important aspects for you when choosing a career.**

| - Financial income |
| --- |
| - Reputation |
| - Possibility of part-time work |
| - Doctor-patient relationship |
| - Primarily performing medical activities |
| - Career opportunities |
| - Autonomy at work |
| - Job security |

***12. With regard to the most attractive career goal selected above [*4], how would you rate the influence of the following factors on your choice of this career goal?**

| **Factor** | **Strong influence against** | **Rather influence against** | **No influence** | **Rather influence in favor** | **Strong influence in favor** |
| --- | --- | --- | --- | --- | --- |
| Opinions and advice from friends | ○ | ○ | ○ | ○ | ○ |
| Opinions and advice from the family | ○ | ○ | ○ | ○ | ○ |
| Needs of society (e.g. shortage of skilled workers) | ○ | ○ | ○ | ○ | ○ |
| Clinical courses with patient contact during your studies | ○ | ○ | ○ | ○ | ○ |
| Lectures/events without patient contact during your studies | ○ | ○ | ○ | ○ | ○ |
| Experiences during the elective study year | ○ | ○ | ○ | ○ | ○ |
| Events outside of the study program (if none were attended, please skip) | ○ | ○ | ○ | ○ | ○ |
| Personal mentoring/advice from doctors or professors | ○ | ○ | ○ | ○ | ○ |
| COVID-19 pandemic | ○ | ○ | ○ | ○ | ○ |
| Political context | ○ | ○ | ○ | ○ | ○ |

**4) Views on medical training and the profession**

***13. Irrespective of your career goals, do you think that your medical studies have prepared you adequately for entering this professional field?**

| **Occupational field** | **Not at all** | **Rather no** | **I do not know** | **Rather yes** | **Yes, surely** |
| --- | --- | --- | --- | --- | --- |
| Primary care in a general practice | ○ | ○ | ○ | ○ | ○ |
| Primary care in a pediatrics or gynecology practice | ○ | ○ | ○ | ○ | ○ |
| Specialized medicine in a practice (e.g. cardiology, gastroenterology, rheumatology, etc.) | ○ | ○ | ○ | ○ | ○ |
| General internal medicine in a hospital (diseases of the internal organs without surgical activities) | ○ | ○ | ○ | ○ | ○ |
| Specialized medicine in a hospital (including surgical specialties) | ○ | ○ | ○ | ○ | ○ |
| Research and teaching at a university/college (academic career) | ○ | ○ | ○ | ○ | ○ |
| Research and development in the private sector | ○ | ○ | ○ | ○ | ○ |
| Other non-clinical or non-medical professions | ○ | ○ | ○ | ○ | ○ |

**5) Information about yourself and your future plans**

***14. Which gender do you identify with?**

- female
- male
- diverse
- no answer

***15. Your age in years:** *(free text field)*

***16. Do you intend to complete a doctoral dissertation after your medical studies?**

- yes
- no
- uncertain

***17. What is your first career plan after medical school?**

- *(Drop-down list: Primary care in a general practice, Primary care in a pediatrics or gynecology practice, Specialized medicine in a private practice, General internal medicine in a hospital, Specialized medicine in a hospital, Research and teaching at a university/college, Research and development in the private sector, Other non-clinical or non-medical professions, I don't know/no career plan yet)*

**Conclusion**

*Thank you for your participation!*
