# Supplementary material for: Dynamics of career attractiveness and preferences among Swiss medical students: an observational study at the end of the master’s program
Source: Med Educ Online. 2025 Nov 30;30(1):2592434. doi: 10.1080/10872981.2025.2592434 (PMC12667345; doi:10.1080/10872981.2025.2592434)
Supplement: Supplementary Material — 2. [file ZMEO_A_2592434_SM4856.docx]

**Supplementary Tables and Figures**

**Figure 1.**  Attractiveness of career options at the end of the master’s program in Swiss medical education (n=364). **Notes:** Answers to question 3 in Likert-scale (see Supplementary Material 1) were reported. The right side showed the percentages of positive responses (rather attractive/very attractive). In the middle were the percentages of neutral responses, and on the left were the percentages of negative responses (very unattractive/rather not attractive).


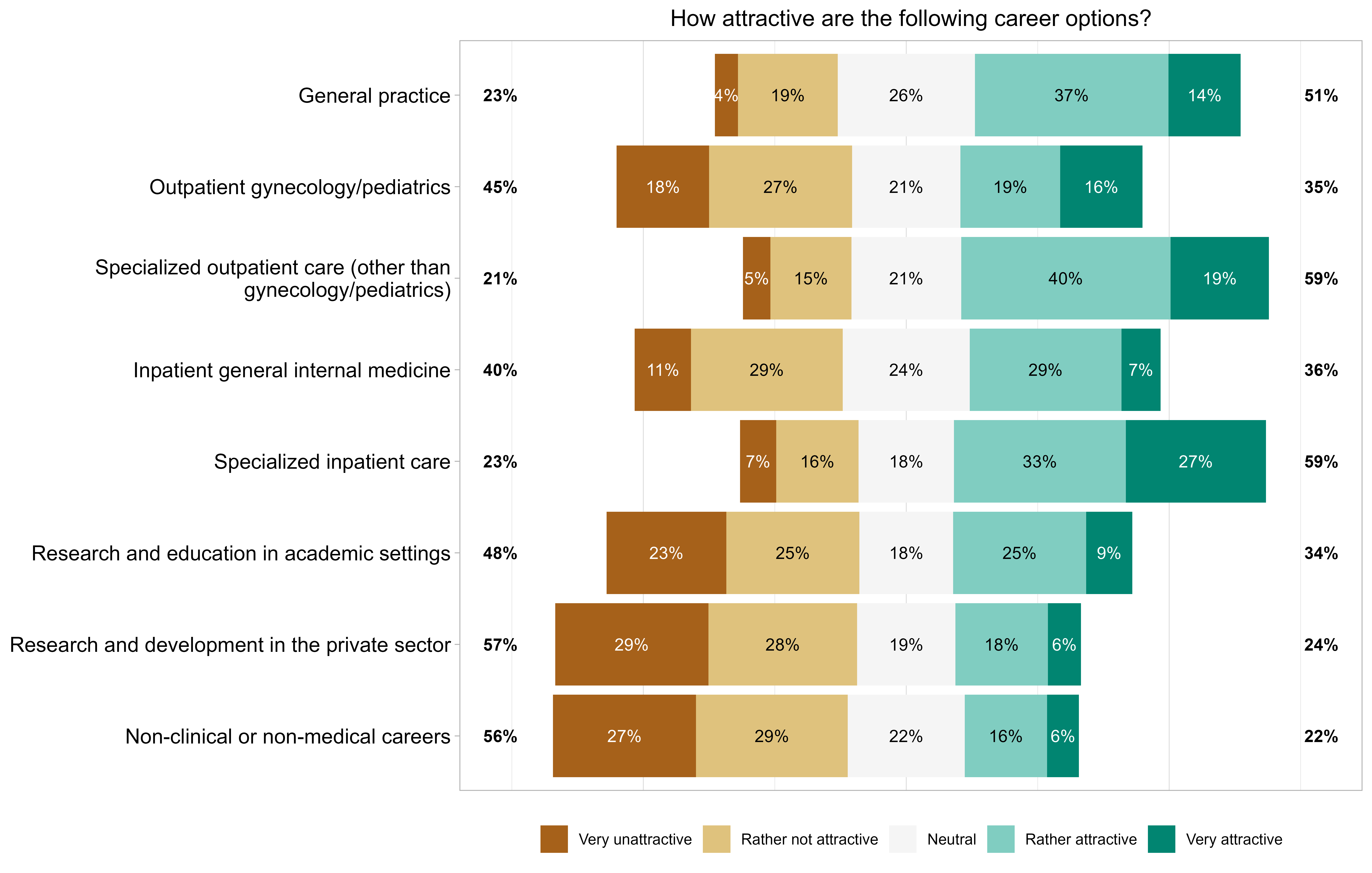


**Table 1.** Qualitative analysis of the free-text answers (see question 5 in Supplementary Material 1). Examples of conceptual codes, identified with tags (second column) separated by a comma, and students’ quotes (first column), were reported.

| **Why is general practice the most attractive career option for you?** | **Tags** |
| --- | --- |
| Autonomy, desire for independence, contribution to the shortage of general practitioners | Autonomy / Independence, Shortage / utility for society |
| Wide range of patients, long-term patient care, possibly more flexibility than in a hospital | Wide range of patients / clinical pictures, Long-term / continuity of care, Flexibility |
| Patient relationships, broad spectrum, working hours | Patient relationships, Wide range of patients / clinical pictures, Working hours / no shifts / part-time |
| Because I would like to have a life and a family alongside my work, and I don't want to work shifts. I also like the long-term care of patients. | Work-life balance, Working hours / no shifts / part-time, Long-term / continuity of care |
| I like working as a GP from the internships and there are many pragmatic arguments in favor of it: part-time, free weekends / evenings, team can be determined by yourself, possible close to home, no dependence on superiors (who can change) | Working hours / no shifts / part-time, Autonomy / Independence |
| **Why is outpatient gynecology/pediatrics the most attractive career option for you?** | **Tags** |
| Would like to work in a practice because of the long-term patient relationship on the one hand and the work-life balance on the other. I think the mix between lots of healthy check-ups and sick consultations is great, which would make it less of a HA practice. | Long-term / continuity of care, Work-life balance, Variety |
| likes children, good working hours. exciting job | Children, Working hours / part-time, Challenge |
| Gynecology: Interface/compromise between a regulated, self-determined everyday life and practicing specialized medicine of my choice at a hospital (occupancy system) | Specialization, Variety, Work conditions / environment |
| Independence (determination of working hours, working models, focus of medical treatments) Clinic can be a very negative working environment (lots of overtime (unpaid), no recognition, sometimes terrible error culture or outdated procedures) Better work-life balance | Autonomy / Independence, Working hours / part-time, Work conditions / environment, Good remuneration, Work-life balance |
| Practice pediatrics to strengthen primary care, compatible with other goals in life and family, attractive working day and climate. Exciting, broad task, varied. Long-term support for children and families possible. | Work-life balance, Primary care, Work conditions / environment, Challenge, Variety, Long-term / continuity of care |
| **Why is specialized outpatient care (other than gynecology/pediatrics) the most attractive career option for you?** | **Tags** |
| Better work-life balance than in a hospital - more freedom to organize your own working hours | Work-life balance, Working hours / no shifts, Autonomy / Independence |
| Work-life balance, good remuneration | Work-life balance, Good remuneration |
| At some point I would like to no longer be directly tied to the hospital and have the opportunity to work independently. Gastroenterology or ENT would be potential opportunities. | No hospital, Autonomy / Independence, Specialty |
| I can decide for myself how much I want to work, what services I want to offer and can go on vacation flexibly. There will be fewer emergencies and no shift work or night shifts. | Autonomy / Independence, Flexibility, No emergency, Working hours / no shifts |
| Free time, good salary, no unfriendly chief physicians, no unpaid overtime, no underpayment and exploitation, having a family is possible | Work-life balance, Work conditions / environment, Good remuneration, Work-life balance |
| **Why is inpatient general internal medicine the most attractive career option for you?** | **Tags** |
| Diverse, complex, interdisciplinary collaboration, exciting | Variety, Complexity, Challenge, Teamwork |
| Versatile, interesting, nice team, training opportunities, part-time work possible | Versatility, Interest, Teamwork, Training, Working hours / part-time |
| Good work-life balance to salary ratio | Work-life balance, Good remuneration |
| Varied, exciting, broad knowledge, contact with different people | Variety, Knowledge, Challenge, Relationships |
| Interest in the subject, possibly later change to a GP practice. | Interest |
| **Why is specialized inpatient care the most attractive career option for you?** | **Tags** |
| The opportunity to gain a lot of experience and different clinical pictures at a specialized center is attractive to me | Wide range of clinical pictures, Specialty |
| Cardiac surgery: professional and technical expertise, i.e. above all surgical activity, with rapid turnover/change of patients, and no excessively old patients. | Surgery / manual work, No patient relationship |
| Specialist in psychiatry with patient contact in the clinic. Would like to practice high-quality medicine in a team. Possibly combine this with research and teaching. | Specialty, High-quality medicine, Teamwork, Research / Teaching |
| I enjoy it the most, it fascinates me the most. Also exciting cases, emergencies etc. Less bureaucracy than in general internal medicine. | Challenge, Emergency, No administrative tasks / bureaucracy |
| For me, doing something manual and working in a specialized way also means dealing with one thing in great detail, in contrast to internal medicine, where you work in a very broad way. | Specialty, Surgery / manual work |
| At the moment, it suits my interests best. It's exciting to work in a team and to keep my finger on the pulse of new research. It's also interesting because there are many exciting polymorbid cases in the center hospital. | Interest, Teamwork, Knowledge, Wide range of clinical pictures |
| **Why is an academic career the most attractive career option for you?** | **Tags** |
| I actually want to do research with a clinic. I appreciate the variety of clinical work (or patient encounters), generating new knowledge and passing on my knowledge. | Variety, Generating knowledge |
| Research interests me and good work life balance | Interest, Work-life balance |
| Because I enjoy research and teaching and I don't like the clinical routine alone. | Interest, No clinical work |
| I want to be able to make a difference in medicine. With this goal in mind, I am freer to organize my working hours, even though I won't be working fewer hours. | Generating knowledge, Make a difference, Autonomy / Independence |
| **Why is research and development in the private sector the most attractive career option for you?** | **Tags** |
| Exciting work, more room for creativity and innovation (entrepreneurship), better working conditions | Challenge, Creativity, Innovation, Entrepreneurship, Work conditions / environment |
| Personal interest in research. Compared to universities, a more secure job with funding. In contrast to clinical work, more work-life balance, more flexible, more part-time options and easier to plan. | Interest, Secure job, Work-life balance, Flexibility, Working hours / part-time |
| Exciting research questions can be pursued. The private sector also offers much more attractive jobs (incl. paternity leave, share dividends, home office, less time pressure). | Challenge, Benefits, Home-office, Less pressure |
| **Why is a non-clinical or non-medical career the most attractive career option for you?** | **Tags** |
| Enables you to work on solving systemic problems, e.g. as a hospital director in the medium term | Solving problems |
| Because the working conditions in the clinic are (usually) inadequate. | Work conditions / environment |
| Working hours, innovation, thinking along, contributing, money, free time, flexibility, hierarchy, remote work, not just guidelines, opportunity to make a difference | Working hours, Innovation, Thinking, Good remuneration, Work-life balance, Flexibility, Hierarchy, Home office, No guidelines, Make a difference |

**Table 2.** Qualitative analysis of the free-text answers (see question 7 in Supplementary Material 1). Examples of conceptual codes, identified with tags (second column) separated by a comma, and students’ quotes (first column), were reported.

| **Why is general practice the least attractive career option for you?** | **Tags** |
| --- | --- |
| I would actually like to become a general practitioner, but I hope that a lot will change in the next 10 years. The effort and responsibility (both towards patients and employees) and the workload are enormous, and the financial remuneration is very poor compared to other disciplines. Unfortunately, it is also very difficult to get assistant positions in various specialist areas such as rheumatology or dermatology, as these are usually only given to “insiders,” which in turn makes it very difficult to get the thorough training required for the skills needed in a family doctor's practice. Although the family doctor curriculum already exists in some hospitals, there are far too few places and even there, promises are often not kept, so that it is not possible to do the relevant rotations. | High responsibility, High workload, Lower financial remuneration, Difficult to get assistant positions in specialized areas, No rotations |
| No interest | No interest |
| Too much psychological care and too little high-tech medicine. | Psychological support, Low high-Tech Medicine |
| A significant proportion (for example) of coughs or similar conditions that are not very exciting from a therapeutic point of view. Relatively poor pay, many patients who take up a lot of time and overload the practice (without adequate grounds for treatment), simply underfunded, even though it should be the cornerstone of medical care (which is why primary care medicine (quality) suffers). | Boring / not challenging, Lower financial remuneration, Patient overloading |
| Doesn't suit my personality, would be too boring/not enough action for me | Boring / not challenging, No interest |
| Administrative work for the practice, less variety compared to the clinic | Administrative tasks, Less variety |
| **Why is gynecology/pediatrics the least attractive career option for you?** | **Tags** |
| I am less interested in specialist areas. | Specialization |
| I'm not interested in pediatrics or gynaecology. I also do a lot of assembly line work, constant stress with what is actually simple work. | No interest, Stress |
| Pediatrics is unattractive for me, both in terms of content and remuneration. Gynecology falls partly into a partially surgical field, which is much more attractive in the practice and in my opinion does not fall into the same category as pediatrics. | No interest, No good remuneration, Relationship with pediatrics |
| Requires a lot of patience, and you earn little. Only women as patients | Patience, Special patient population, No good remuneration |
| Children are not my thing and I don't find gynaecology exciting either | Special patient population, No challenge |
| Mainly related to gynecology, I have already completed 2 internships abroad in the field of gynecology and also did a month of gynecology in Switzerland during my Uhu time. I had great teams and good experiences everywhere. However, I also realized that this specialist area would not be my career goal. | No interest |
| **Why is specialized outpatient care (other than gynecology/pediatrics) the least attractive career option for you?** | **Tags** |
| Very boring and very specialized | Boring, Specialization |
| I'm not interested enough in the individual subjects to only do these | No interest |
| **Why is inpatient general internal medicine the least attractive career option for you?** | **Tags** |
| Inefficient processes, a lot of bureaucracy for nothing, an unpredictable daily routine, few part-time positions, a constantly changing team, little flexibility, too few staff. | Inefficiency, Bureaucracy, Working hours, Rotation, Little flexibility, Work conditions / environment |
| It often feels like you're not achieving very much. The long visits and deliberations and back and forth don't seem very satisfying to me. Lots of chatter, lots of documentation, lots of consultations without getting anywhere | Administrative tasks, Inefficiency, Not rewarding |
| I am less interested in the problems, especially the fact that patients are often not cured because they are chronically ill. | No interest, Chronic / old / multimorbid patients |
| Bad experiences in the WSJ. Hardly any patient contact, but a lot of paperwork and administration. | Bad experience, No patient relationships, Administrative tasks |
| Because I simply don't think the working conditions and pay are right compared to other specializations (reward crisis). In addition, you have all the disadvantages of working in a hospital (poor occupational benefits, no predictable daily routine due to shift work, and little time with/for patients). | No good remuneration, Work conditions / environment, Working hours, Rotation, No patient relationships |
| **Why is specialized inpatient care the least attractive career option for you?** | **Tags** |
| Bad work-life balance. A lot of stress. Not enough appreciation and remuneration for the work done. | No work-life balance, Stressful, No appreciation / remuneration |
| Underpaid, no appreciation, no compliance with labor law, unpaid overtime and no life of their own anymore | No autonomy, Working hours, Work conditions / environment, No work-life balance |
| Surgery because it's a terrible lifestyle and most people are wasting away under these circumstances. | Surgery / manual work |
| Only regarding surgery: no work-life balance, still too patriarchal and hierarchical | Surgery / manual work, No work-life balance, Work conditions / environment |
| I definitely don't want to go into surgery. Too much competition, too long working days, emergency services, no private life, fewer opportunities to become self-employed | Surgery / manual work, Working hours, Work conditions / environment, Emergency, No work-life balance, No self-employment |
| **Why is an academic career the least attractive career option for you?** | **Tags** |
| Because I like being in direct contact with people and not studying medicine for 6 years and then doing research instead of working clinically. | No clinical / medical work, No patients |
| I'm not interested enough in research, I'd rather work with other people/patients. | No interest, No patients |
| I like having defined areas of responsibility, I find research work difficult because you have to define your thesis yourself and have a duck to get a publication or something similar. | Define your work / objectives, Pressure to results / publication |
| Had little contact with it during my studies | Little experience |
| What I enjoy about medicine is the contact with people and the feeling of being able to make a direct impact. That gets a little lost in research. | No patients |
| Does not suit me | No interest |
| **Why is research and development in the private sector the least attractive career option for you?** | **Tags** |
| Too many defeats before a small success. In the private sector additionally too economically/profit-oriented, in contrast to university, where it is a bit more about progress in science. | Profit-oriented, Too many efforts |
| Don't know much about it, sounds boring, no patient contact | Boring, No patients, No knowledge about it |
| A healthcare system serves the general population and not private greed for profit. That would not be compatible with my values. | Profit-oriented |
| Not interested in research. Private sector sometimes ethically difficult, and research per se of course too | No interest, Profit-oriented |
| not interesting enough for me and don't know enough about private sector research | No interest, No knowledge about it |
| Research and development is not the reason why I studied. I want to have contact with patients and the bureaucracy behind research is not so attractive to me. | No interest, No patients, Bureaucracy |
| **Why is a non-clinical or non-medical career the least attractive career option for you?** | **keywords** |
| Because my heart beats for the clinic and the people :) | No clinical / medical work, No patients |
| Because I am still satisfied with my studies/career prospects. | Satisfaction with studies |
| Because it has nothing to do with my studies, which correspond to my interests | No other interest, No relevance with studies |
| I am studying medicine to become a doctor. As this is my absolute dream job, doing something else is out of the question for me. | No clinical / medical work, No other interest |
| I chose this course because I like working with people and helping people. This is less the case in a non-clinical profession. | No patients |
| I really enjoy working in a medical profession. Therefore, I have no interest in pursuing non-clinical career goals. | No clinical / medical work, No other interest |

**Figure 2.** Tag cloud of reasons for selecting the most attractive career option (see question 4 and 5, Supplementary Material 1). **Notes:** Tags were the ones reported in Table 1 above. The percentage for each tag was reported in relation to the n students answering the question. The number of missing values in relation to students who expressed a preference for the career was also reported.

**
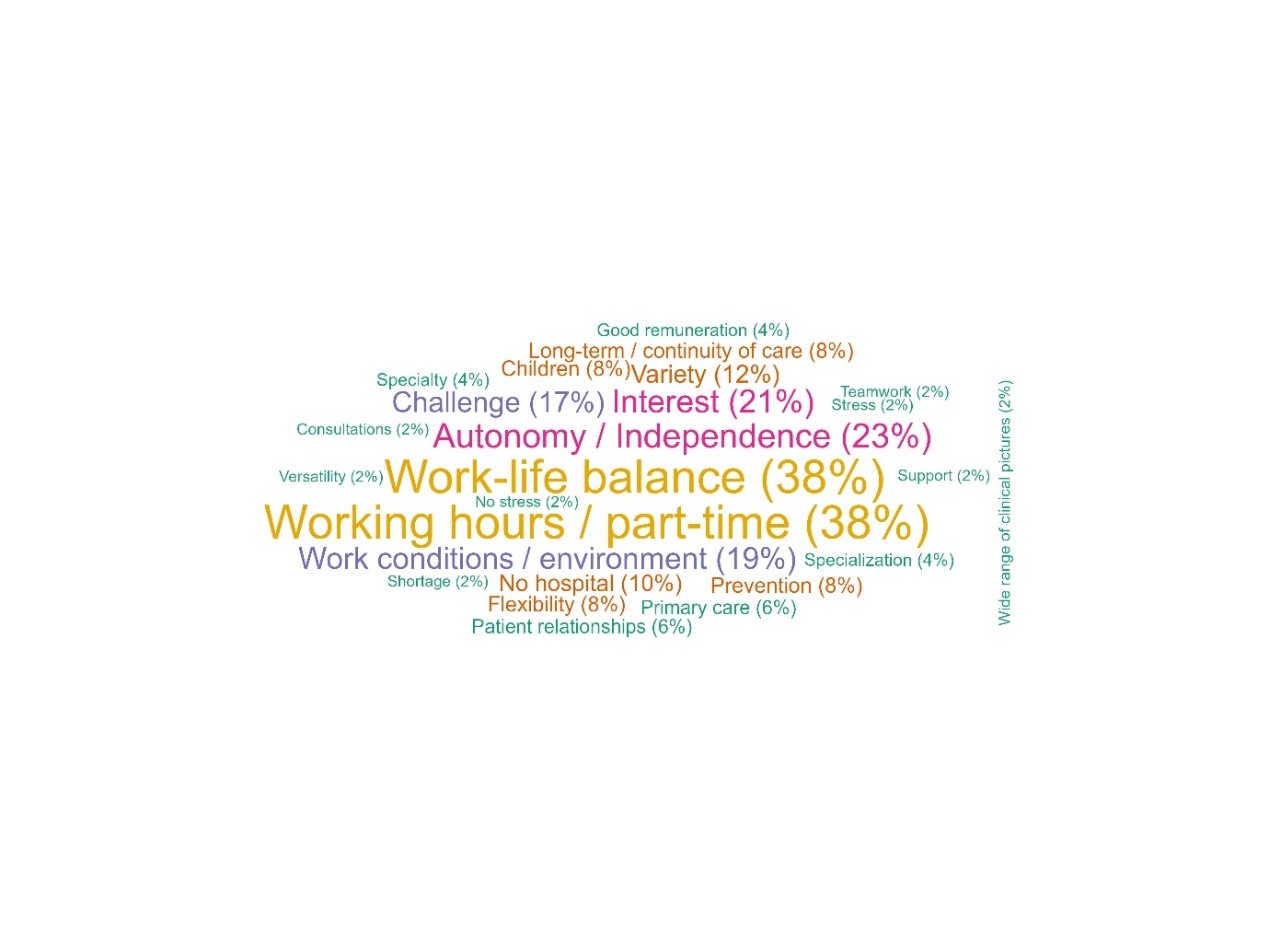

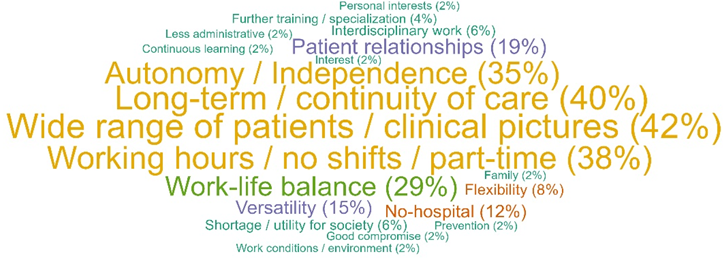
**

**a) General practice** (n=48, missing=3)

**b) Outpatient gynecology/pediatrics** (n=48, missing=4)

**c) Specialized outpatient care (other than gynecology/pediatrics)**

(n=61, missing=12)

**d) Inpatient general internal medicine** (n=13, missing=2)

**
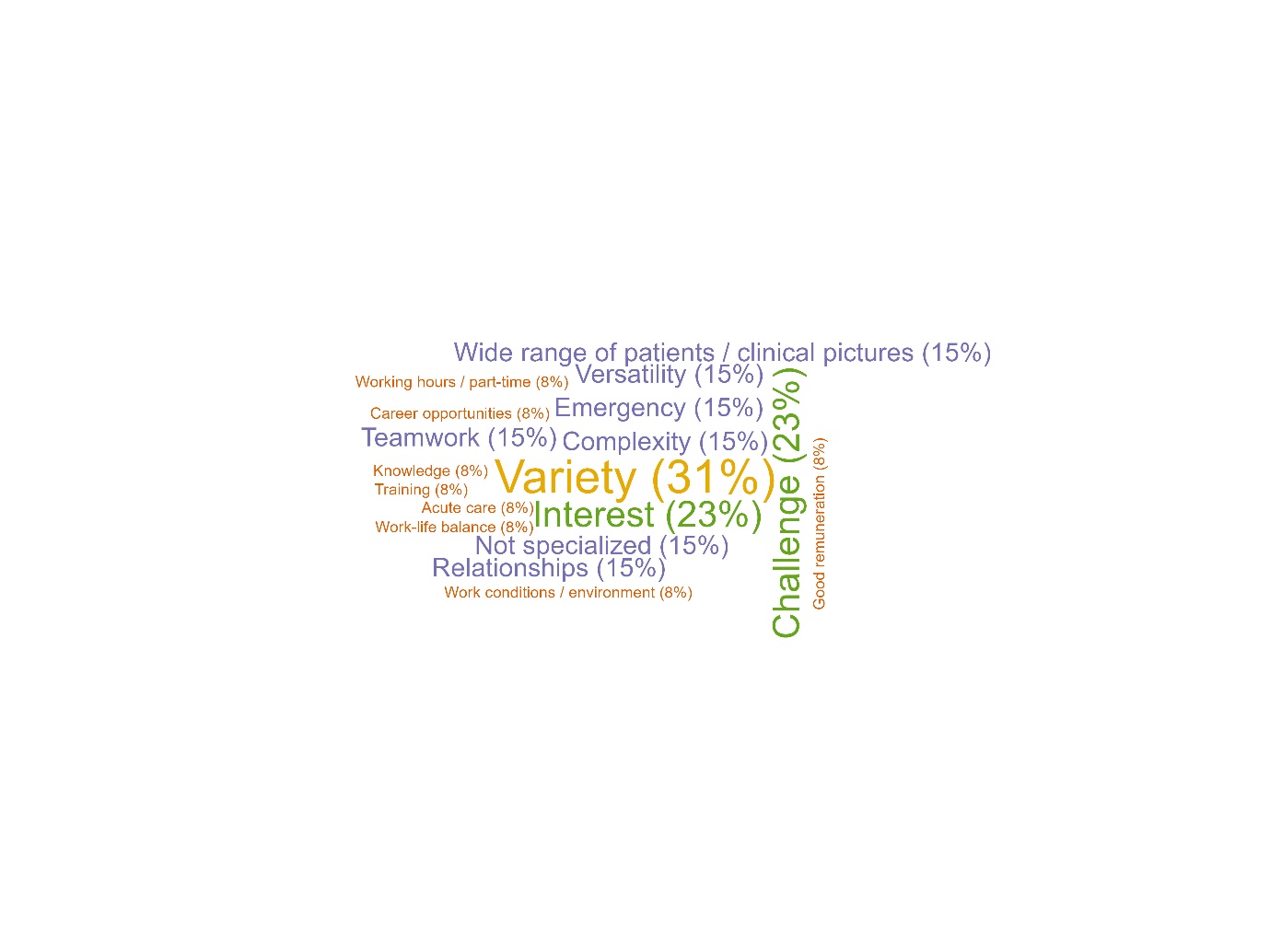
**
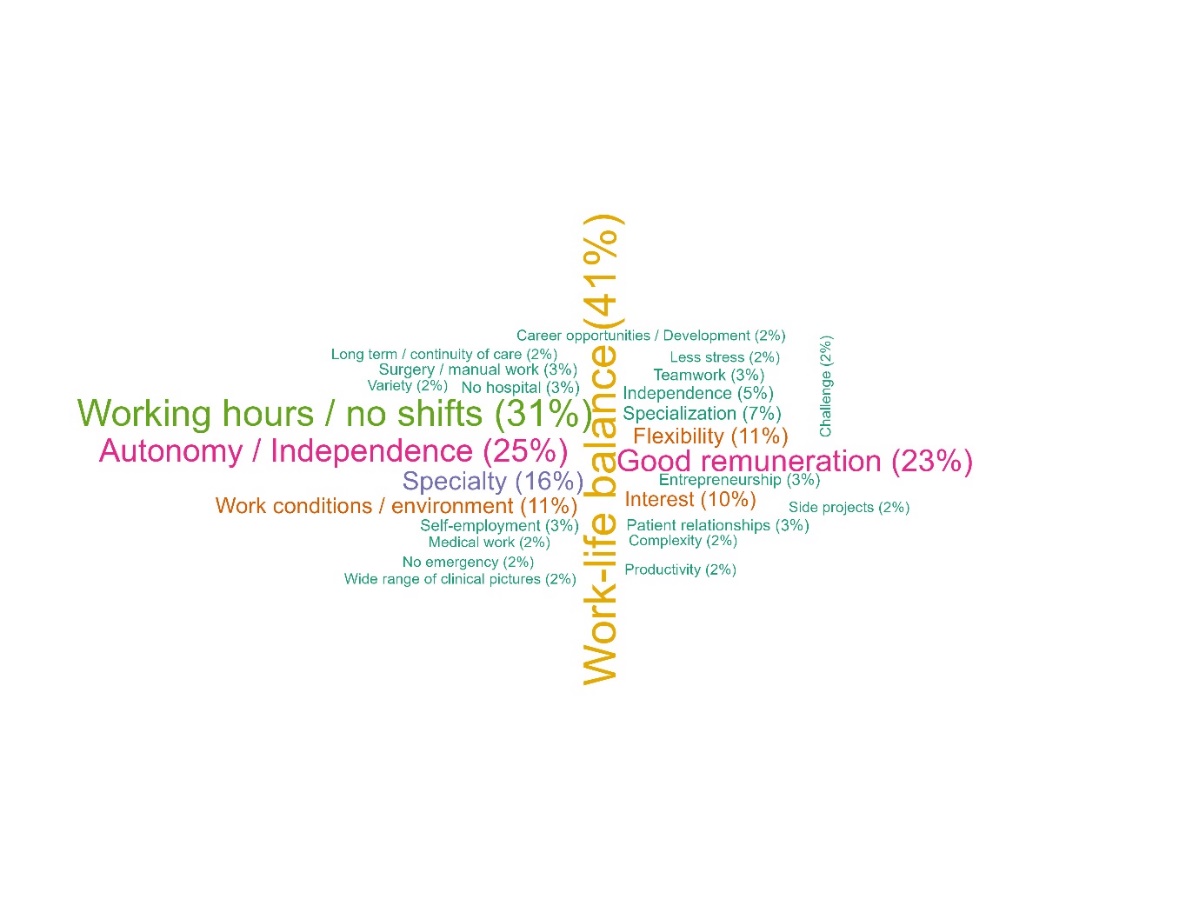


**e) Academic career** (n=9, missing=2)

**g) Non-clinical or non-medical careers** (n=5, missing=1)

**
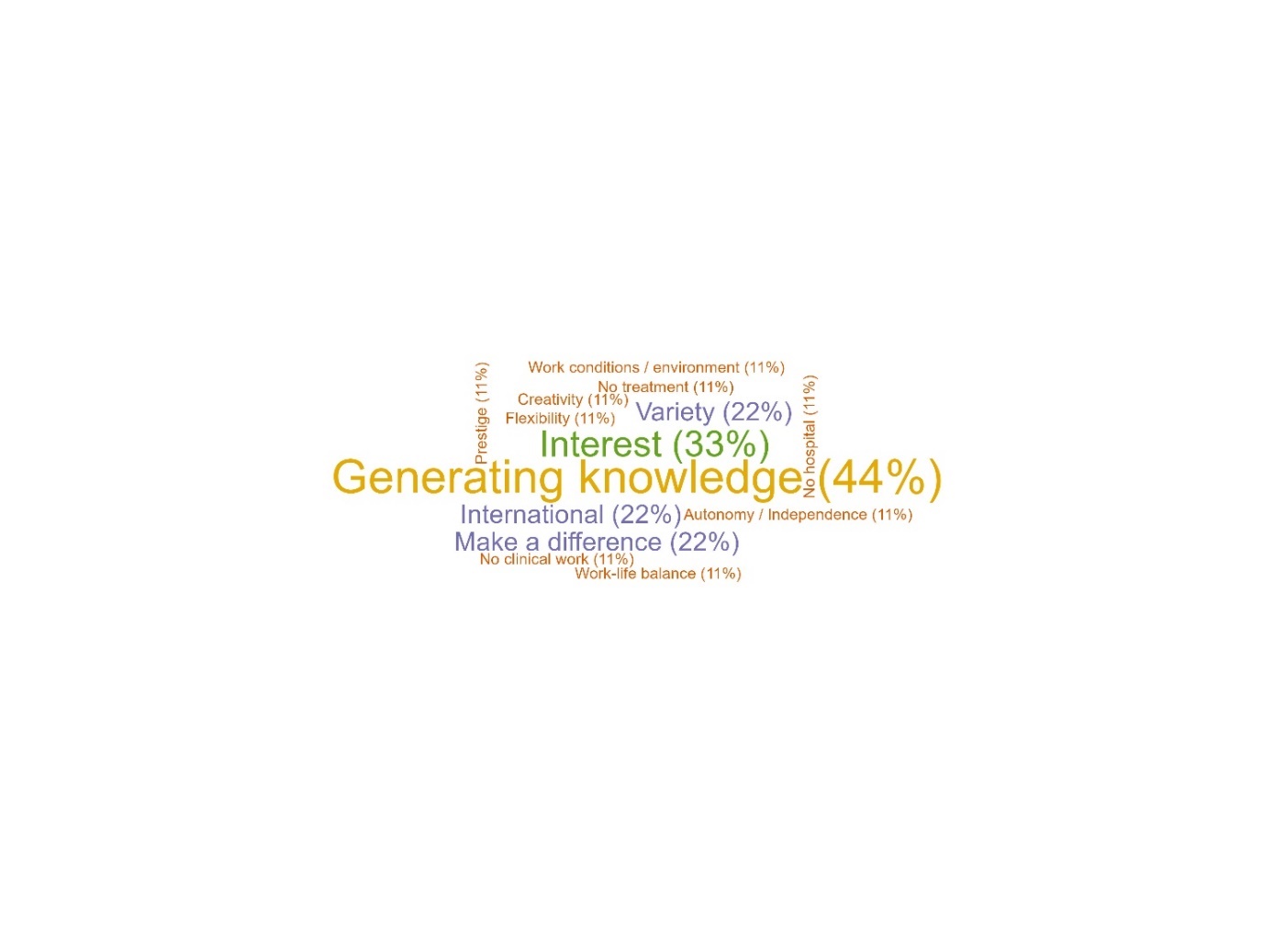
**

**f) Research and development in the private sector** (n=7)

**
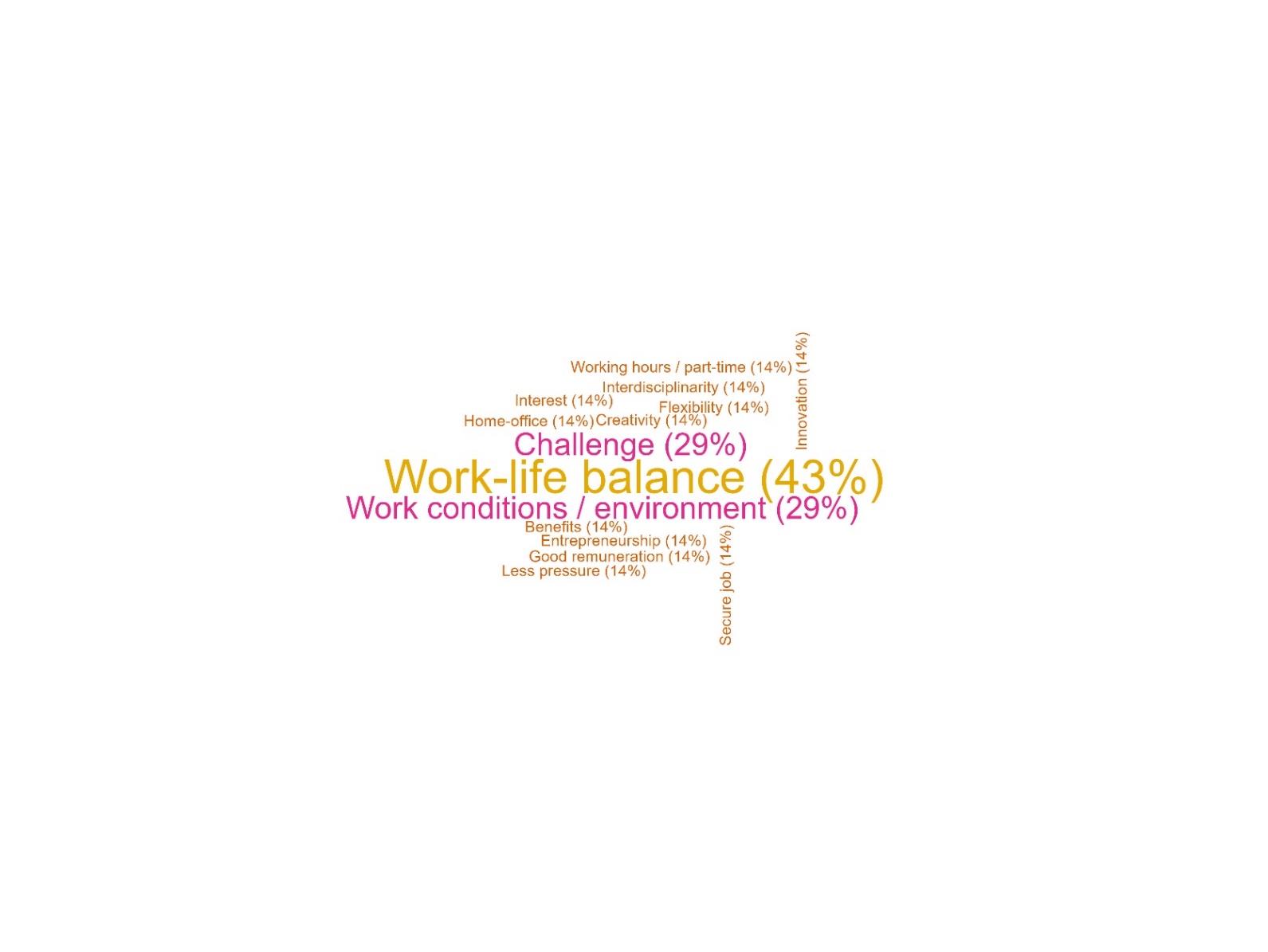

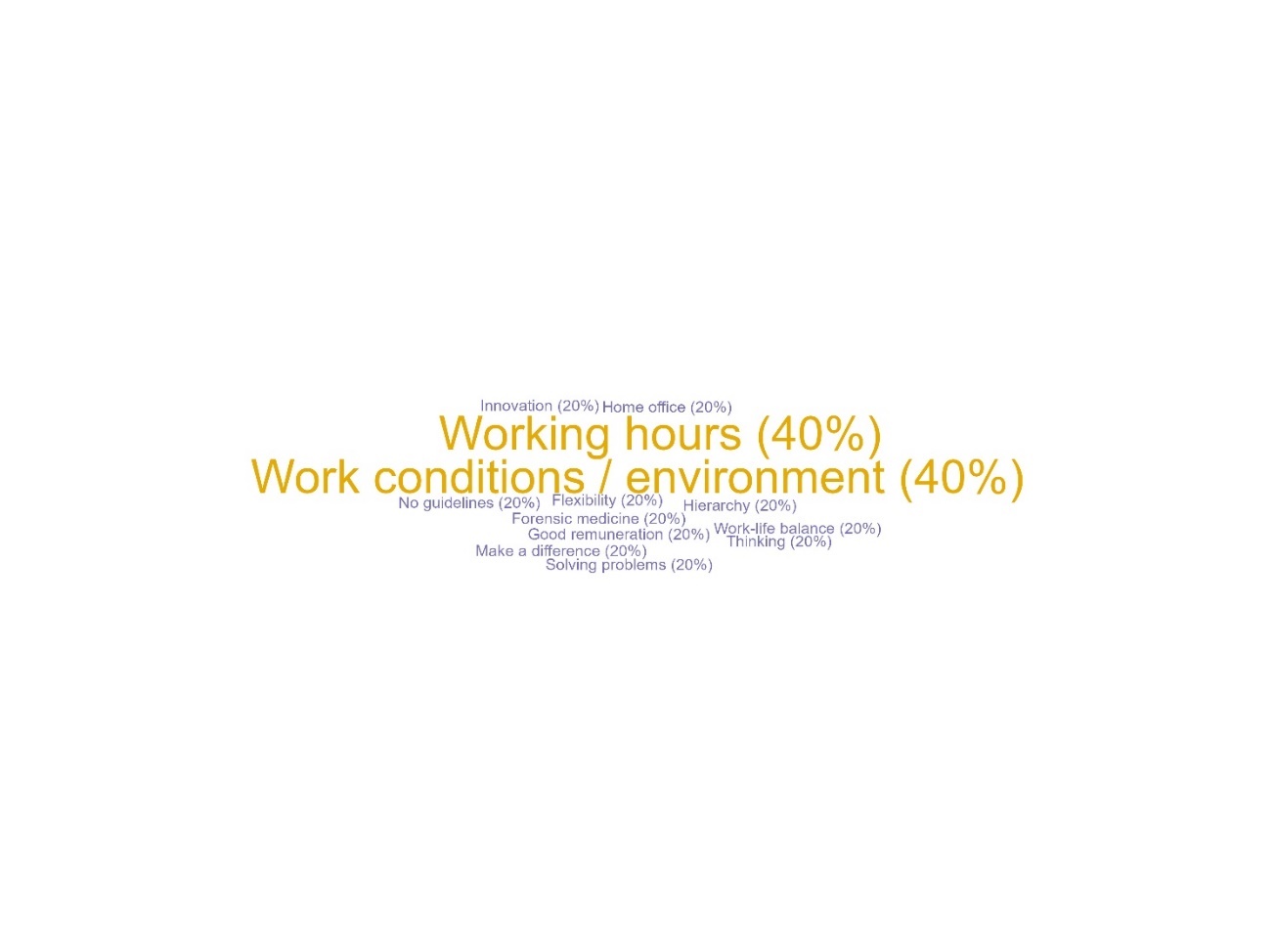
**

**Figure 3.** Tag cloud of reasons for selecting the least attractive career option (see question 6 and 7, Supplementary Material 1). **Notes:** Tags were the ones reported in Table 2 above. The percentage for each tag was reported in relation to the n students answering the question. The number of missing values in relation to students who expressed an opting out of the career was also reported.

**b) Outpatient gynecology/pediatrics** (n=50, missing=7)

**a) General practice** (n=14, missing)
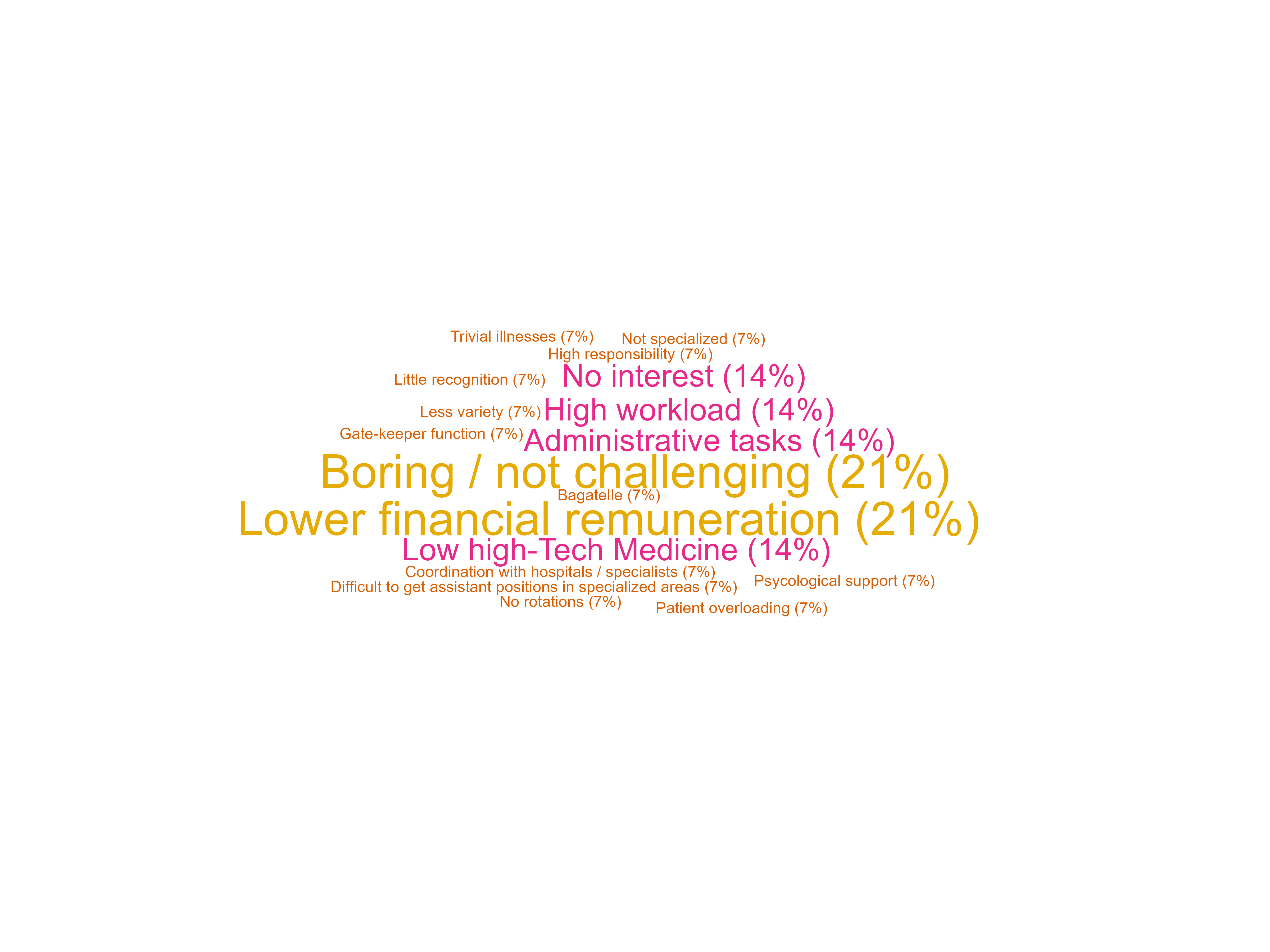


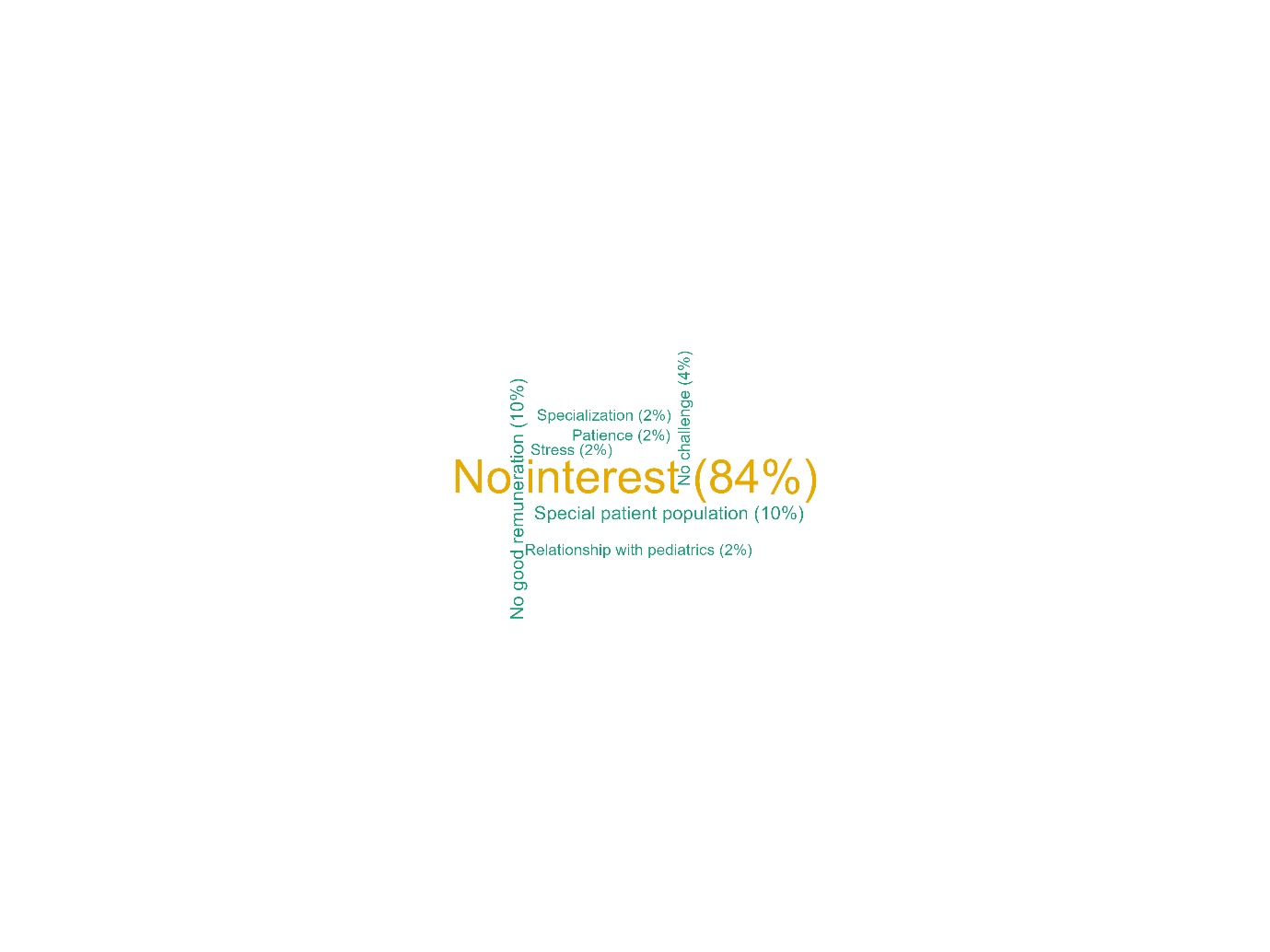
**
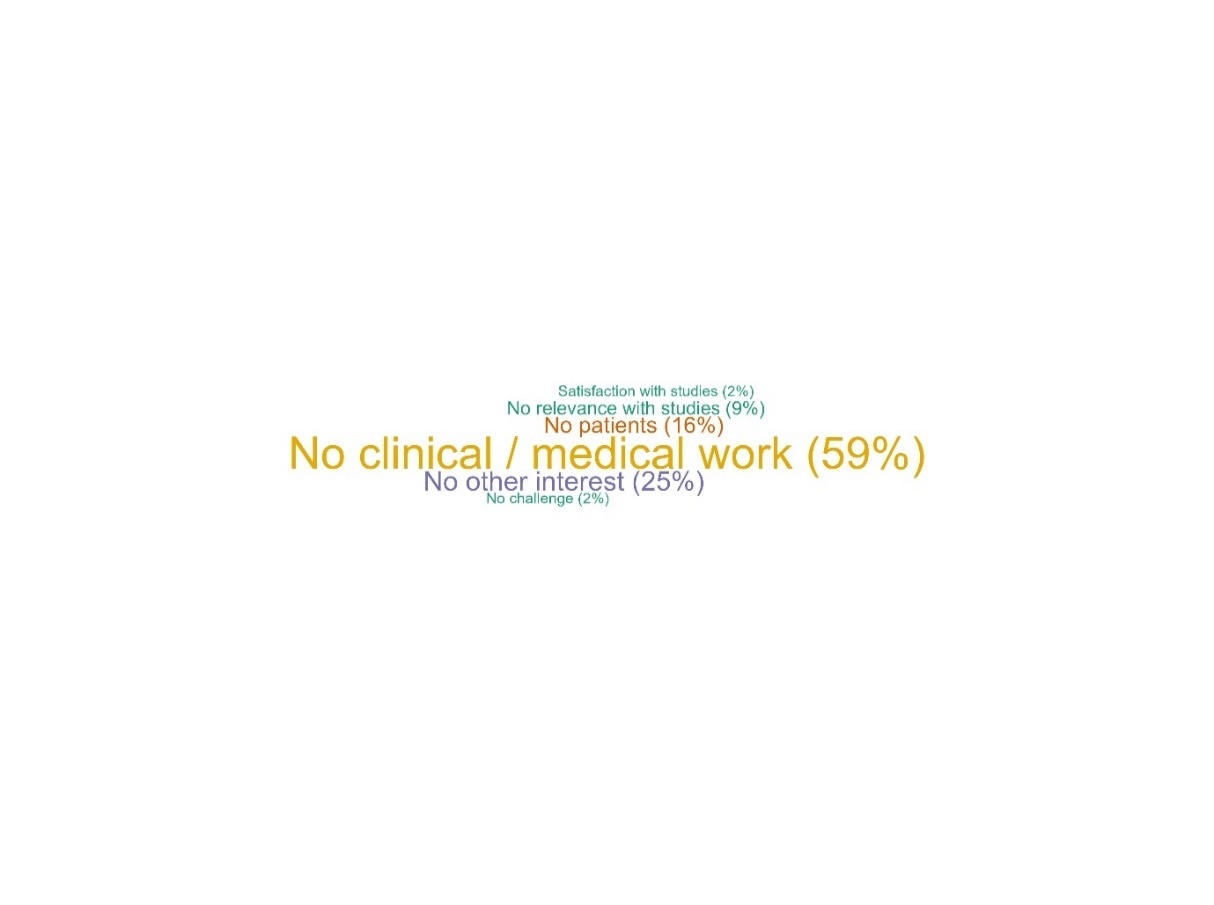

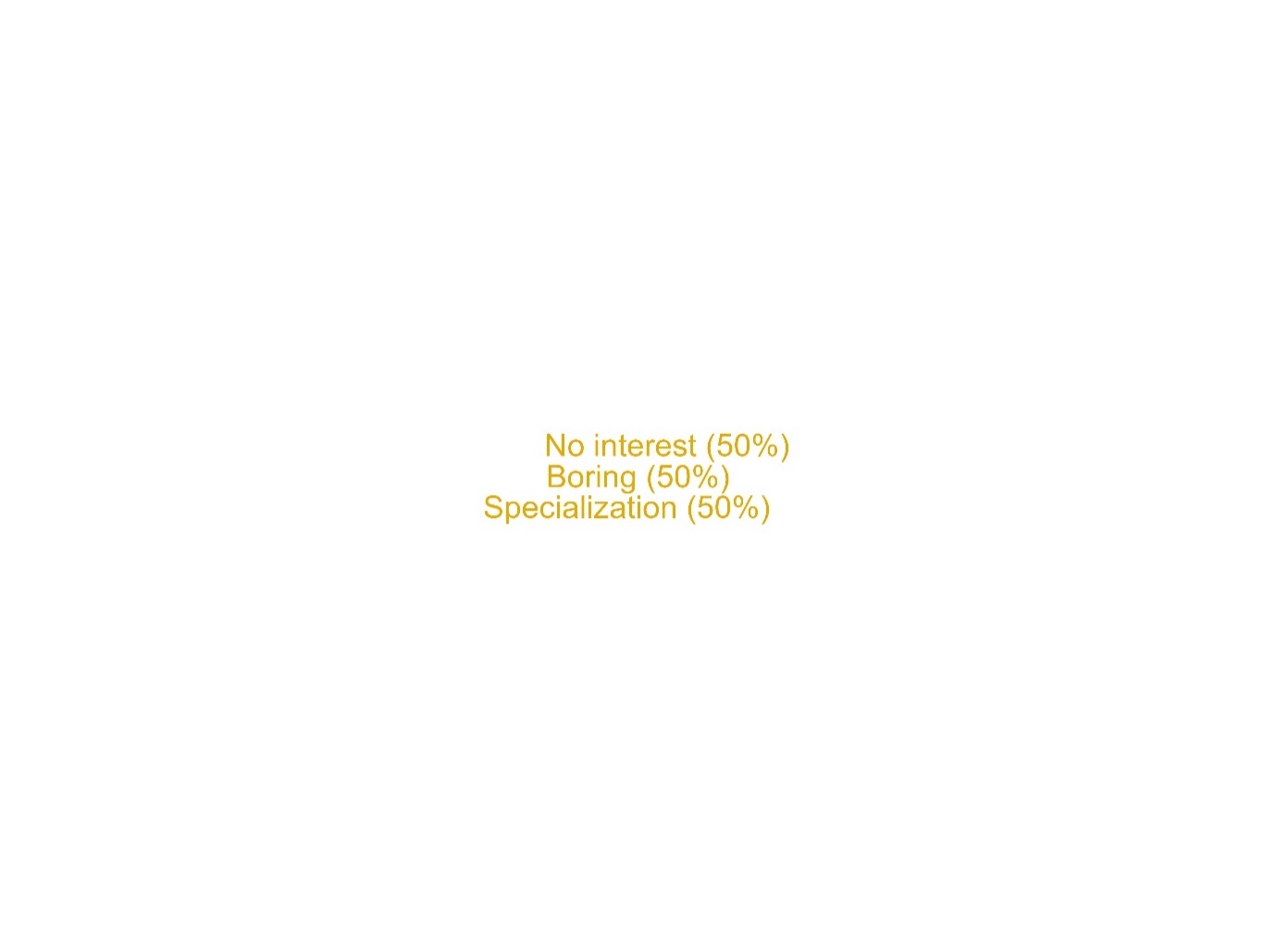
**

**d) Inpatient general internal medicine** (n=43, missing=6)


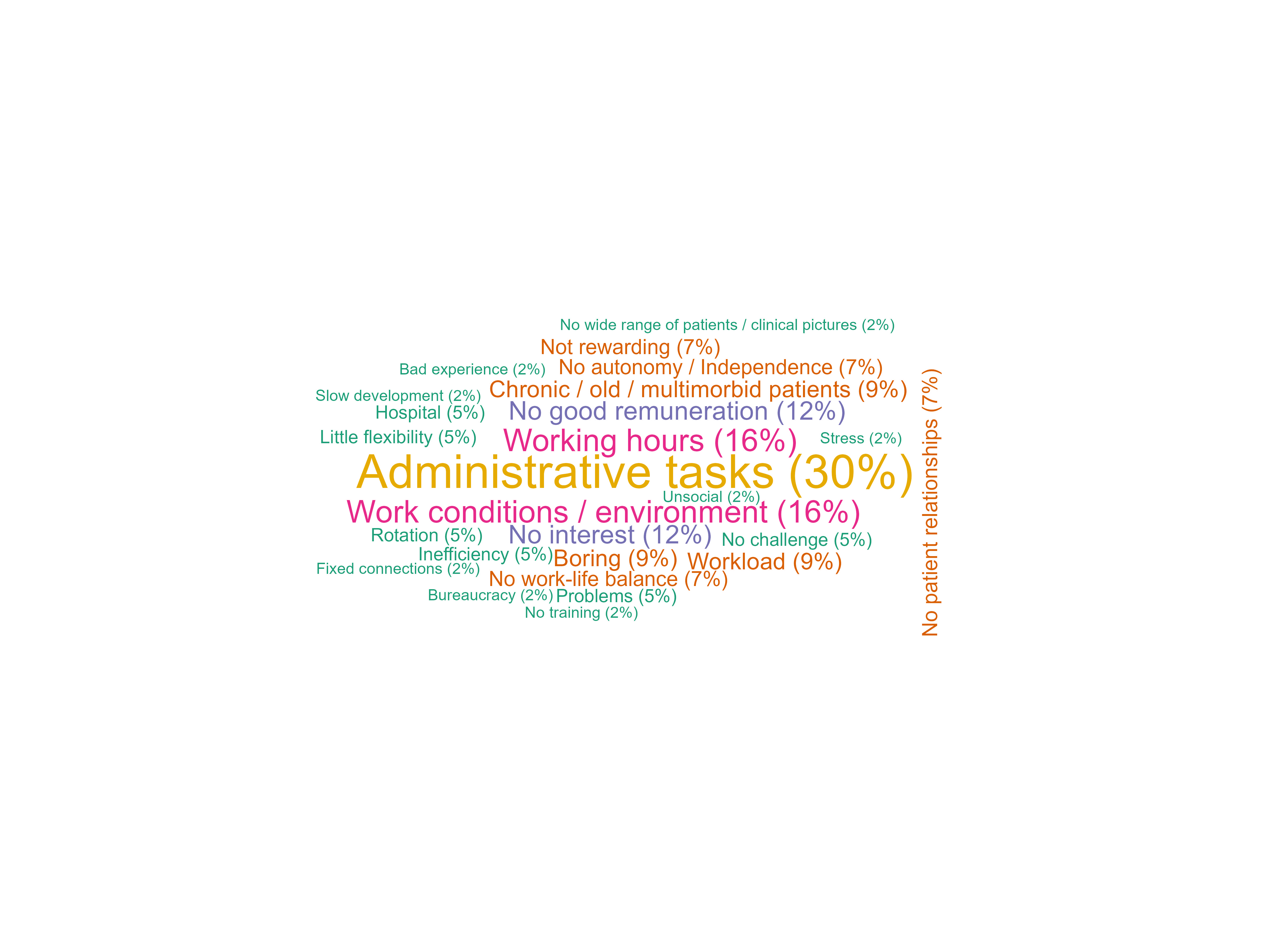


**e) Specialized inpatient care** (n=33, missing=2)


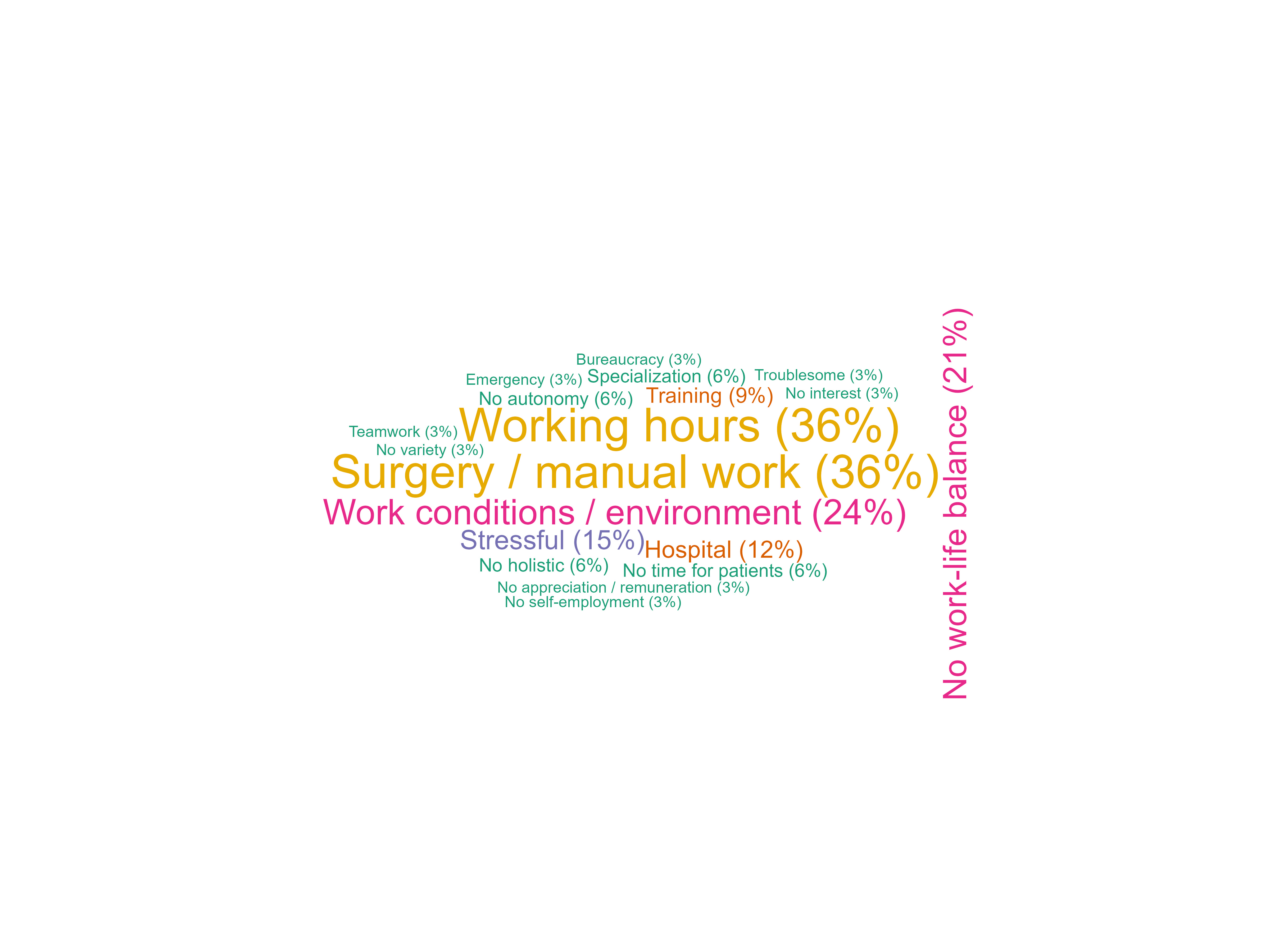


**g) Research and development in the private sector** (n=49, missing=9)


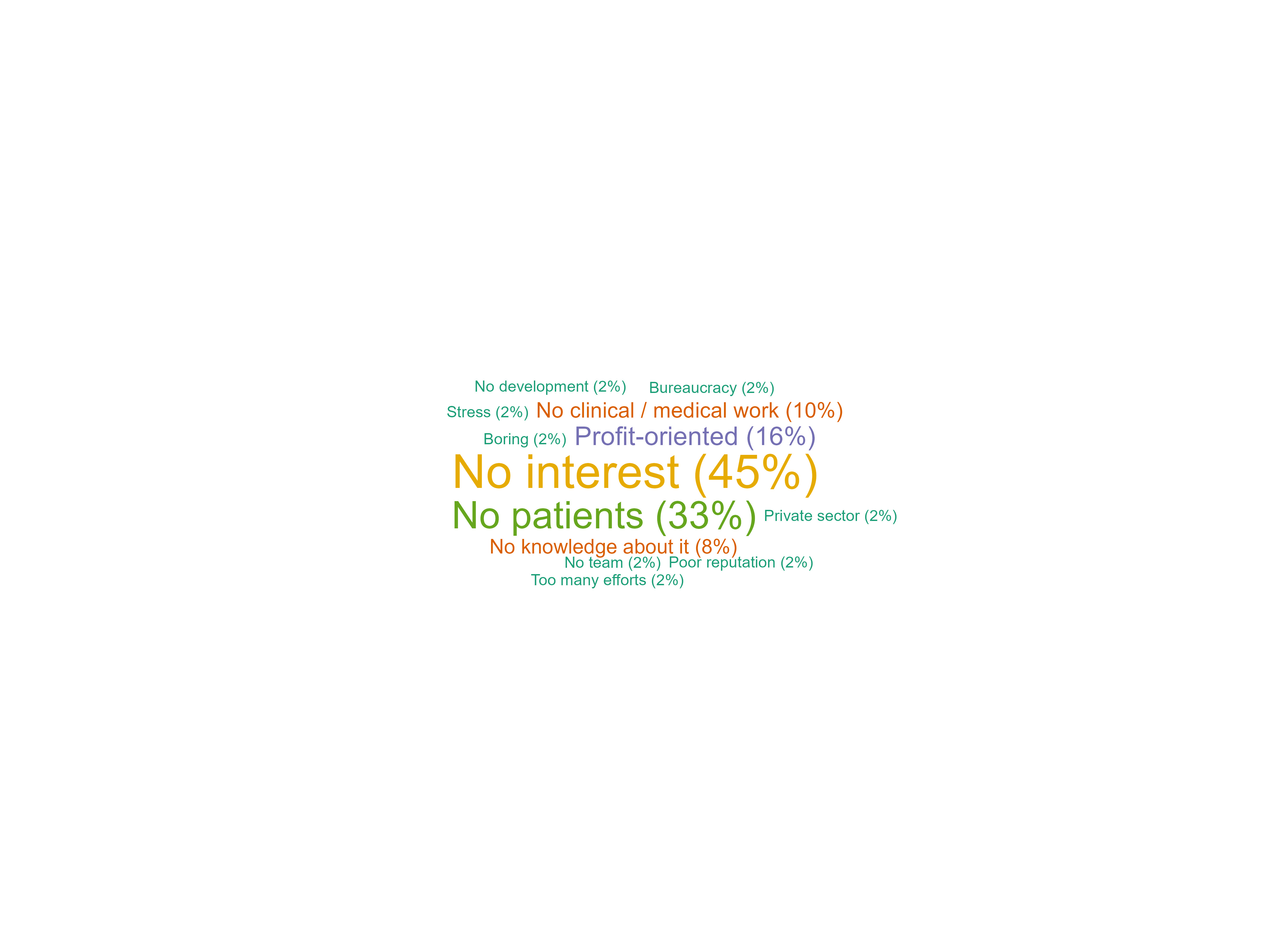


**f) Academic career** (n=43, missing=10)


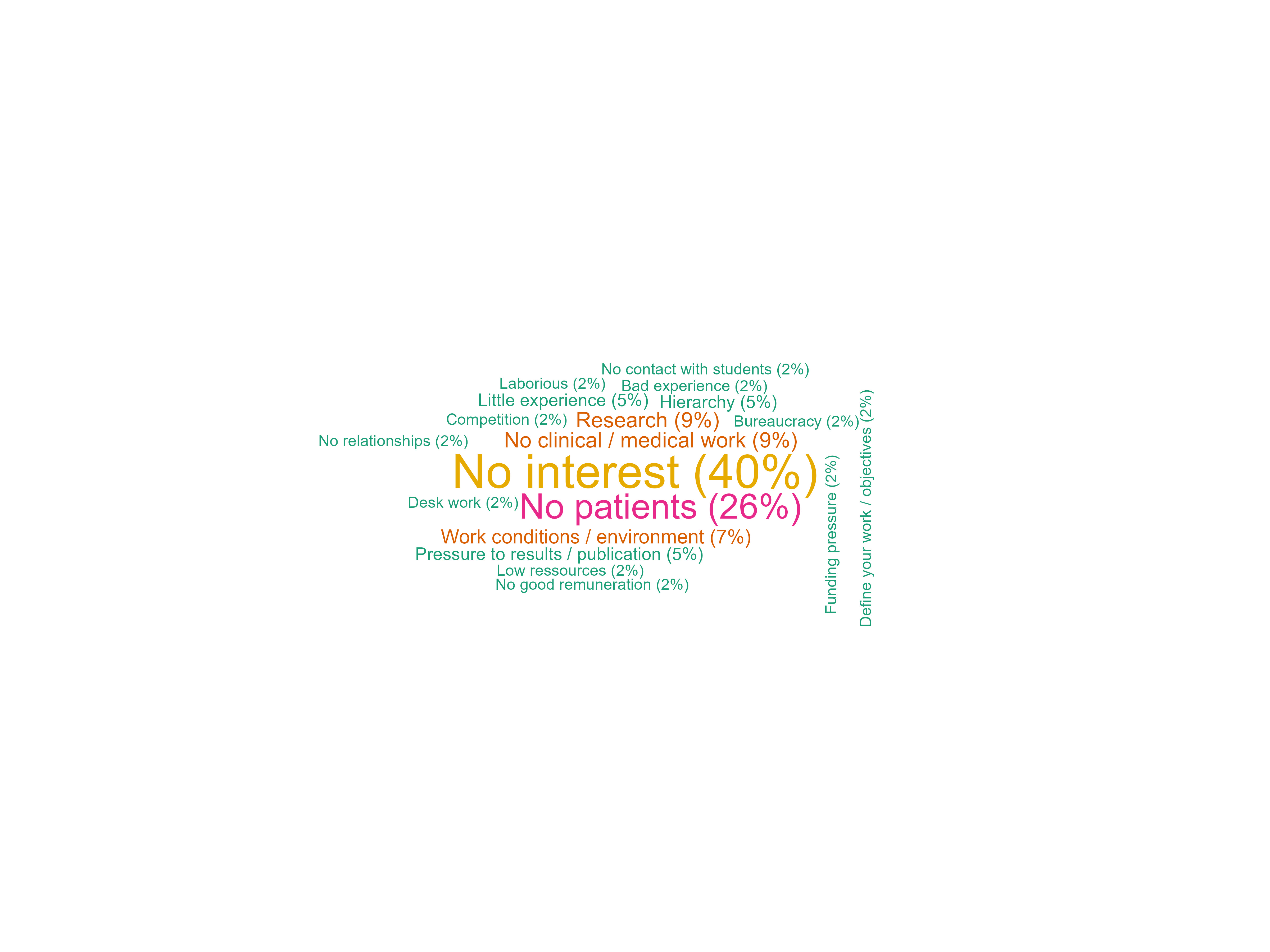


**h) Non-clinical or non-medical careers** (n=56, missing=7)

**c) Specialized outpatient care (other than gynecology/pediatrics)** (n=2, missing=2)



**Figure 4.** Alluvial plot of the students’ dynamics of the attractiveness of each career option during medical education. **Notes:** The first and second dimensions were the changes in attractiveness during the bachelor’s and master’s program, respectively (see questions 8 and 9 in Supplementary Material 1). The third dimension was the career preference at the end of medical education (question 4) recoded as follows: yes = selected as the most attractive career option, no = not selected as the most attractive career option. A total of 345 answered all three questions. Percentages of the students in each stratum and flow were reported when percentages were above 1%.

**Figure 5.**  Importance of career determinants at the end of the master’s program in Swiss medical education. **Notes:** Answers to question 10 in Likert-scale (see Supplementary Material 1) were reported (n=344, n=343 for autonomy and job security). The right side showed the percentages of positive responses (rather important/very important). In the middle were the percentages of neutral responses, and on the left were the percentages of negative responses (not at all important/rather not important).


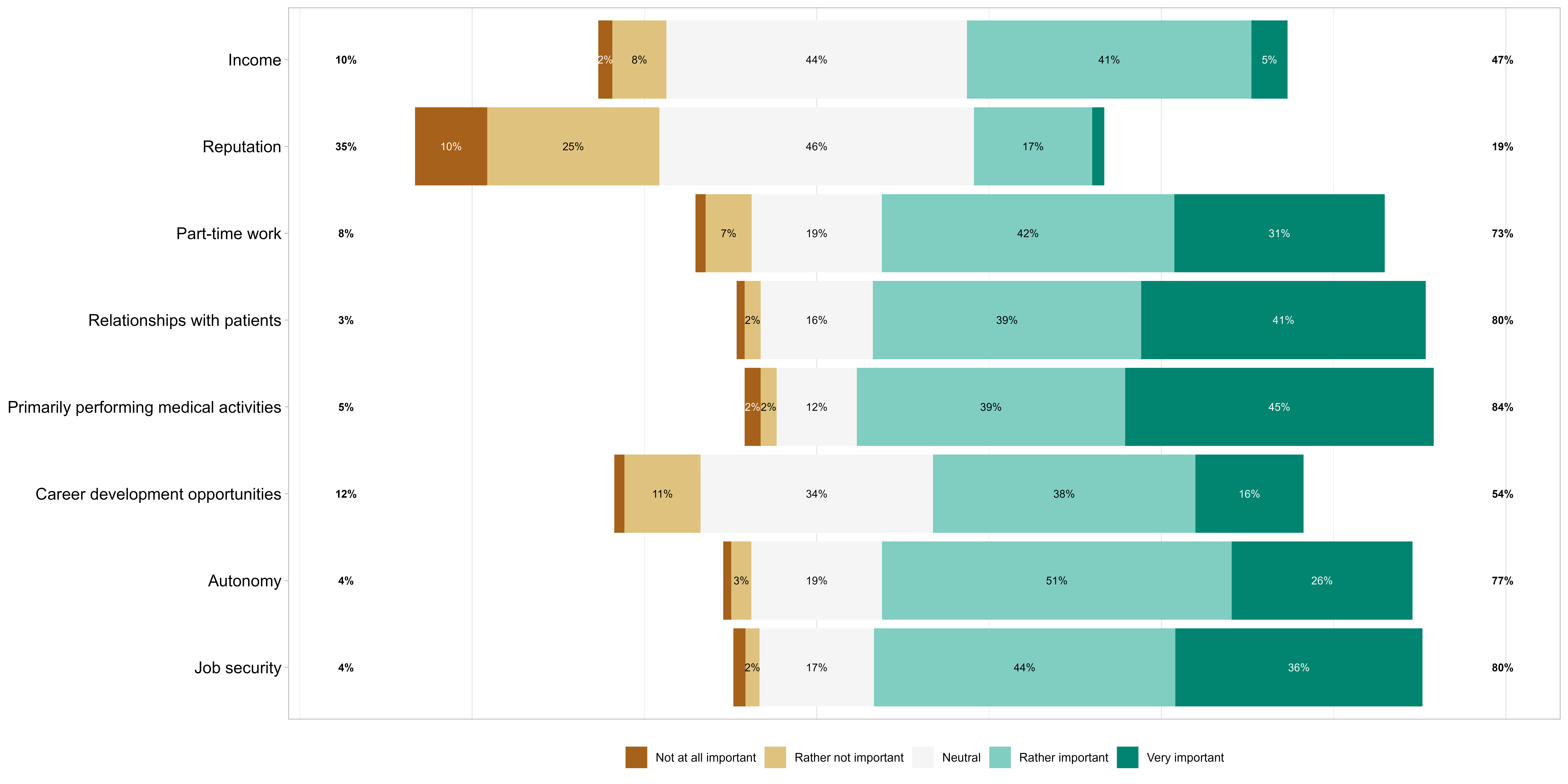


**Figure 6.** Factors favoring or hindering a career choice at the end of the master’s program in Swiss medical education: results stratified by career preference. **Notes:** The results are relative to the responses to question 12 and stratified by responses to question 4 (see Supplementary Material 1). The right side showed the percentages of positive responses (rather influence in favor/strong influence in favor). In the middle were the percentages of neutral responses (no influence), and on the left were the percentages of negative responses (strong influence against/rather influence against).


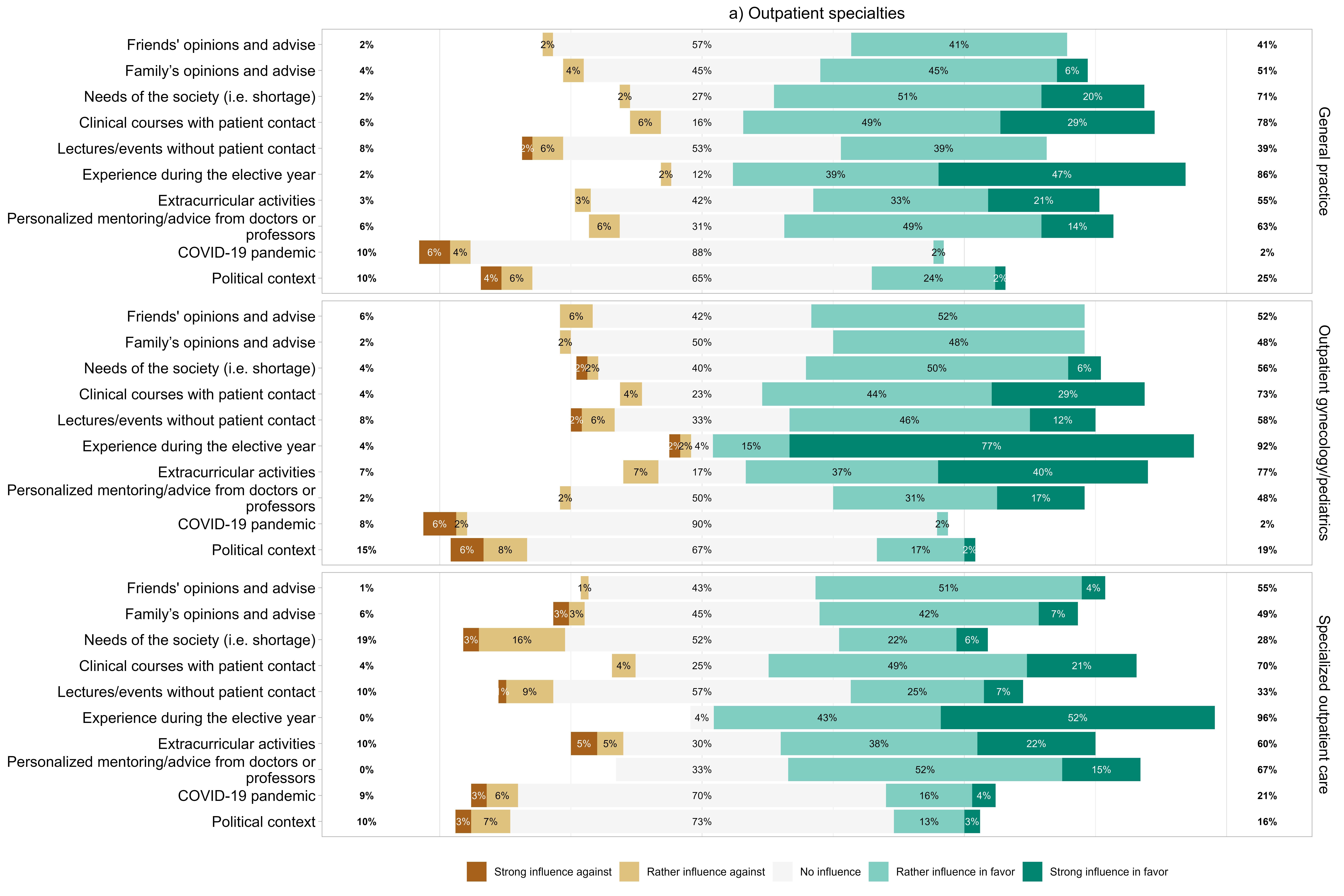


**
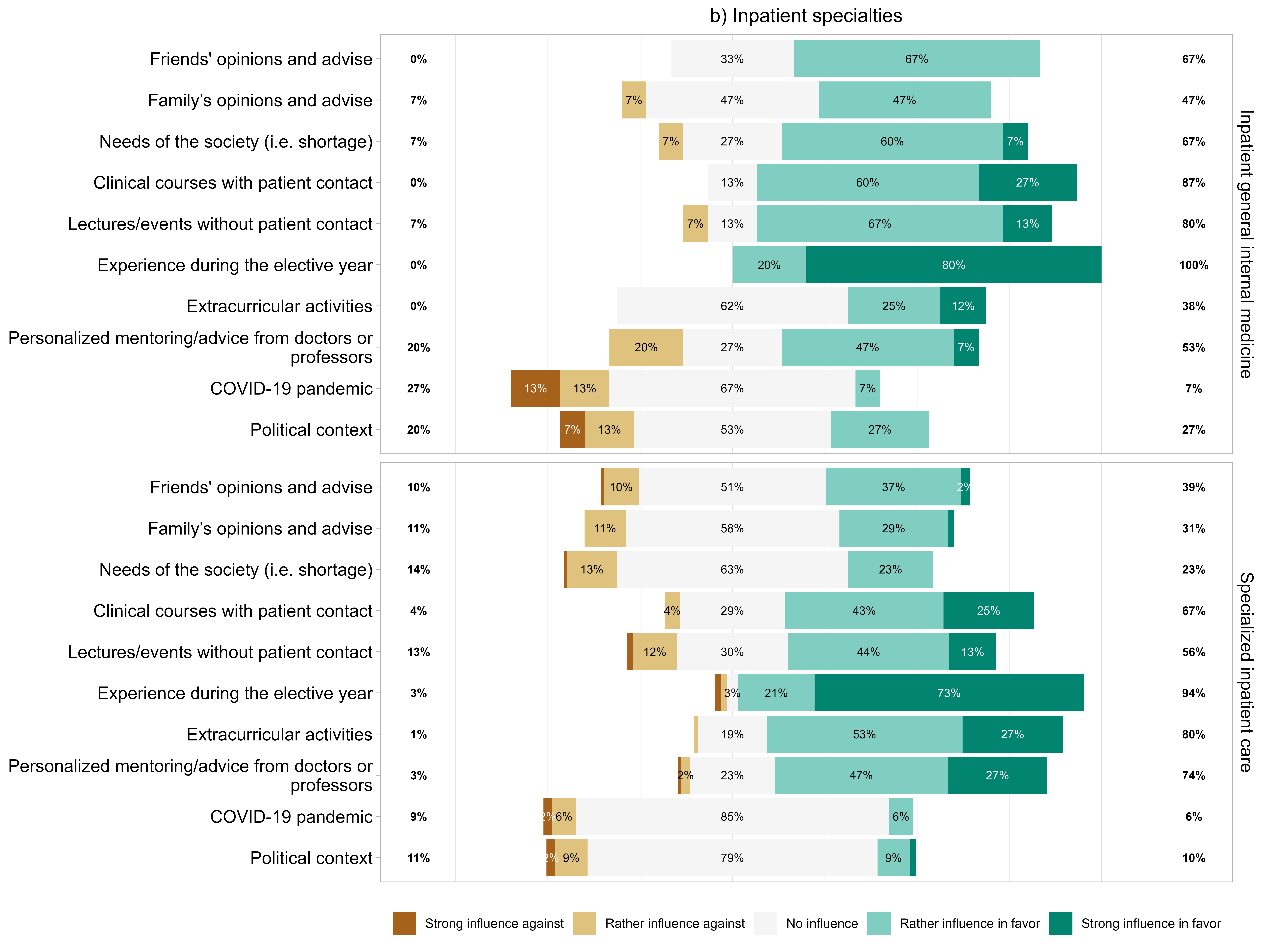
**

**

**
